# Potential Therapeutic and Medicinal Applications of Four Invasive Non-Native Plant Species: A PRISMA-Guided Systematic Review of PubMed Studies

**DOI:** 10.3390/plants14192966

**Published:** 2025-09-24

**Authors:** Ingrid Nădășan, Mihai Babotă, Aura Rusu, Corneliu Tanase

**Affiliations:** 1 Doctoral School of Medicine and Pharmacy, “George Emil Palade” University of Medicine, Pharmacy, Science and Technology of Târgu Mureș, 38 Gheorghe Marinescu Street, 540139 Targu Mures, Romania; 2 Department of Chemistry and Medical Biochemistry, Faculty of Medicine in English, “George Emil Palade” University of Medicine, Pharmacy, Science and Technology of Târgu Mureș, 38 Gheorghe Marinescu Street, 540139 Targu Mures, Romania; 3 Research Centre of Medicinal and Aromatic Plants, “George Emil Palade” University of Medicine, Pharmacy, Science and Technology of Târgu Mureș, 38 Gheorghe Marinescu Street, 540139 Targu Mures, Romania; 4 Department of Pharmaceutical Botany, Faculty of Pharmacy, “George Emil Palade” University of Medicine, Pharmacy, Science and Technology of Târgu Mureș, 38 Gheorghe Marinescu Street, 540139 Targu Mures, Romania; 5 Pharmaceutical and Therapeutic Chemistry Department, Faculty of Pharmacy, “George Emil Palade” University of Medicine, Pharmacy, Science and Technology of Târgu Mureș, 540142 Targu Mures, Romania

**Keywords:** *Ailanthus altissima*, *Amorpha fruticosa*, *Asclepias syriaca*, *Phytolacca americana*, ailanthone, antimicrobial activity, antioxidant activity, cytotoxicity, invasion biology, plant valorisation

## Abstract

Invasive non-native plant species are of ecological concern globally, as they may negatively affect biodiversity, the economy, and human health. At the same time, invasive non-native plants comprise an underutilised biomass that contains valuable natural bioactive compounds, which could find various biomedical applications and potential medicinal uses. In this paper, we aimed to systematically review the published data surrounding four selected invasive non-native plant species in a medical and therapeutic context. The search was conducted using PubMed and PRISMA guidelines, and strict criteria were employed to provide a thorough framework for the study selection process. After rigorous screening of the 53 selected articles, we were able to summarise the main findings and current knowledge regarding the valorisation opportunities for the selected plants in a medical context and to identify research gaps and highlight further research opportunities. Finally, we concluded that the selected invasive non-native plant species may provide valuable services in the biomedical field if the focus of future research is concentrated on their potential applicability in clinical settings. Furthermore, the valorisation of invasive non-native plant species may prove to be a viable strategy for controlling their spread.

## 1. Introduction

### 1.1. The Impact of Invasive Non-Native Plant Species and Their Possible Valorisation

Invasive non-native plant species (INPS) are those plant species that are introduced into a new ecosystem to which they do not naturally belong, either through natural means (wind, water, animals, etc.) or through human activity (directly and indirectly). Some of the introduced plant species may become naturalized in their non-native ecosystems and thus cause no harm; however, others tend to spread uncontrollably, while at the same time causing damage to the local biodiversity (flora and fauna) and disturbances of the soil, potentially causing harm to humans, domestic animals, pets, and the economy. Among the human-assisted factors contributing to the spread of INPS are increased travel and tourism activities, increased global trading, climate change, and intentional or unintentional use of non-native plant species as decorative plants [1,2,3,4,5].

The impact of INPS spreads across multiple domains, and significant efforts are being made to eradicate them. Most countries adhere to action plans for controlling these plant species, which generally aim to reduce the social and economic harm that they cause. The action plans usually consist of educating and involving communities in spotting and reporting newly introduced species, planning out and implementing control management actions at the authority level (through chemical control, uprooting, or controlled fire), and by strictly regulating the handling of these plant species through local, nationwide, or union-level laws [6,7,8,9]. At the European Union (EU) level, the Invasive Alien Species Regulationspecifies a list of strictly regulated non-native species, some of which are considered of Union concern but are not strictly regulated. According to this law, all member states are required to take action to prevent the introduction of new non-native species in their territories, to take measures for early detection and quick eradication of these species, and to find feasible ways to manage the spread of already widely established non-native species in their territories. More importantly, species that are strictly regulated are prohibited from being kept, imported, sold, bred, grown, and/or released into the environment [10].

Conventional methods for the management of the spread of INPS are very costly, sometimes ineffective, and often result in wasted biomass [11,12,13]. Considering that resources in general are limited, management methods such as finding value in INPS by collecting and utilising them through the valorisation of plant biomass could potentially be a more viable spread control alternative [14,15]. This method includes, but is not limited to, the extraction and purification of bioactive compounds with medicinal, therapeutic, and cosmetic applications, transforming the plant mass into various materials (fibres, paper, etc.), livestock feed, bioenergy/biofuel, biochar, compost, and wood products, all of which could contribute to covering at least some parts of the cost of eradication, a concept that is known as the circular economy [16,17,18,19,20,21,22,23]. Most importantly, this approach contributes to a more sustainable, cost-friendly, and more efficient management of INPS.

Plants are recognized as a significant source of bioactive compounds that can be extracted and utilized in the medical, pharmaceutical, and cosmetic fields. Screening plants for bioactive compounds and testing them using various assays has led to essential drug discoveries, supplement formulation, and applications in diverse biological activities, such as antioxidant, anti-inflammatory, antimicrobial, and anticancer uses, to name a few [24,25,26]. Although significant progress has been made in elucidating the therapeutic potential of plant species and the mechanisms of action of their phytochemicals, substantial gaps in knowledge persist. What makes INPS especially interesting to study in this sense is the fact that they are highly adaptable organisms with the ability to spread and compete with the native flora and fauna, and the specific conditions of the ecosystems in which they are introduced is in part due to their chemical composition, which varies based on the location [27,28,29].

### 1.2. Argumentation of the Study

There is growing interest in the ecological management of INPS, but their biomedical potential remains underexplored. Although numerous studies have investigated the phytochemical profiles of different plant species, there is a lack of systematic reviews focused explicitly on the therapeutic applications of INPS. The existing scientific literature tends to highlight ecological control strategies or isolated pharmacological discoveries, without synthesising the broader biomedical relevance of these species. Thus, a comprehensive evaluation of INPS as sources of bioactive compounds with potential therapeutic value is needed, especially in the context of the circular economy and sustainable resource use.

To select the INPS to be included in the literature review, databases and studies focusing on the most abundant and problematic non-native plant species established in Romania were consulted. EU regulations on invasive alien species were also considered during the selection process. The first compiled database of invasive and potentially invasive plant species in Romania was published in 2022. After the authors of the aforementioned study performed a literature review, they identified 102 plant species of concern [30]. Following this, the first-ever nationwide field survey in Romania brought new information regarding the distribution of non-native plant species. It revealed that there are 396 species belonging to 77 families established in Romania, updating the initially published database. Of the invasive plants established and widespread on Romania’s territory, four are also of EU concern: *Ailanthus altissima*, *Asclepias syriaca*, *Elodea nuttallii*, and *Impatiens glandulifera*. The study also proposed a set of seven plants of interest for Romania: *Ambrosia artemisiifolia*, *Ambrosia tenuifolia*, *Ambrosia trifida*, *Cyclachaena xanthiifolia*, *Phytolacca americana*, *Phytolacca acinosa*, and *Verbesina encelioides*, from which three were considered to exert a significant impact and to require even more focused management plans and measures: *Ambrosia artemisiifolia*, *Ambrosia trifida*, and *Phytolacca americana* [31].

The spread of INPS in national parks and protected natural areas poses an even greater challenge, since in these areas, chemical control is strictly prohibited. Several studies have highlighted potentially damaging INPS that are spreading and causing biodiversity loss within these protected areas. Dumitrascu M et al. performed a survey of several protected areas in Romania, including the Danube Delta Biosphere Reserve, the Mureș Floodplain, and Comana Natural Park. They identified *Amorpha fruticosa* as being the INPS with the most negative impact on these protected ecosystems [32].

For the present study, we have selected the following INPS to be included, based on their impact, spread, and possible valorisation opportunities for therapeutic uses as means of managing their population numbers: *Ailanthus altissima*, *Amorpha fruticosa*, *Asclepias syriaca*, and *Phytolacca americana*.

### 1.3. Study Objectives

The primary objective of this systematic review is to critically evaluate the current scientific literature concerning the therapeutic and medicinal applications of four invasive non-native plant species: *Ailanthus altissima*, *Amorpha fruticosa*, *Asclepias syriaca*, and *Phytolacca americana*. The selected INPS, while ecologically problematic, possess underutilised biomass rich in bioactive compounds with potential biomedical value. By synthesising existing research, the study aims to identify and highlight the therapeutic potential of INPS, including their antioxidant and antimicrobial properties, as well as their cytotoxic and neuroprotective effects.

Additionally, the review proposes to uncover gaps in current knowledge and suggest future research directions that could support the sustainable valorisation of these invasive species. Our approach aims to contribute to the advancement of natural product-based therapeutics and to offer an innovative strategy for managing INPS through their biomedical exploitation, aligning with principles of a circular economy and ecological sustainability.

## 2. Methodology

### 2.1. Botanical Terminology

All four botanical names were cross-checked for accuracy with Plants of the World Online (https://powo.science.kew.org/, accessed on 16 June 2025), the International Plant Names Index (IPNI) (https://www.ipni.org/, accessed on 16 June 2025), and the World Flora Online (WFO) Plant List (https://wfoplantlist.org/, accessed on 16 June 2025) [33,34,35]. The native or non-native status of the plants in each study was checked using Plants of the World Online (https://powo.science.kew.org/, accessed on 16 June 2025) [33].

### 2.2. Data Collection

For this systematic review, we followed the PRISMA protocol and guidelines to effectively and accurately identify, screen, and include relevant articles for the chosen topic. Rayyan (Cambridge, MA, USA), an AI-powered web-based platform designed to simplify the process of conducting systematic reviews, was used for article screening [36].

Searches were conducted on PubMed, considering reviews focusing on the medicinal and therapeutic use of the selected plants. The keywords used were the botanical names of the plants included in the study, as follows: “*Ailanthus altissima*”, “*Amorpha fruticosa*”, “*Asclepias syriaca*”, and “*Phytolacca americana*”. The screening process was conducted independently by two reviewers. Discrepancies during the study selection process were resolved through discussion, and when consensus could not be reached, a third reviewer served as an arbitrator.

The quality of the included studies was assessed narratively, considering methodological aspects such as whether the study design was suitable for the stated objectives, the level of detail and reproducibility of methods, the traceability of the sample, how confounding factors were managed, and transparency in reporting the results. No formal validated instrument was used because the primary goal of the analysis was descriptive, aiming to synthesize and contextualize existing evidence without applying a standardized quality classification.

The inclusion criteria for the studies were the following:

1. The cited research must utilise a traceable plant material or isolated phytocompound, with either a deposited voucher specimen or a clearly stated source.
2. The cited articles must be written in English or have an English translation available.
3. Only original research articles were considered; reviews, commentaries, and other non-original formats were excluded.
4. The study must evaluate at least one type of biological activity, conducted either in vitro, in vivo, or both.

### 2.3. Data Analysis

The data obtained during the article screening process were manually recorded and managed using Microsoft Excel for Microsoft 365 (Version 2508). Visualisation of data and graphics was conducted manually using Microsoft Excel for Microsoft 365 (Version 2508).

## 3. Results

### 3.1. Literature Screening

The literature search was performed on 26 April 2025 using PubMed, yielding a total of 1100 articles as follows: for *Ailanthus altissima*, the search provided a total of 408 results; for *Amorpha fruticosa*, a total of 132 results; for *Asclepias syriaca*, a total of 173 results; and *Phytolacca americana*, a total of 387 results. No lower time limit for the publication date was applied, while the upper limit was 26 April 2025.

**Figure 1:**
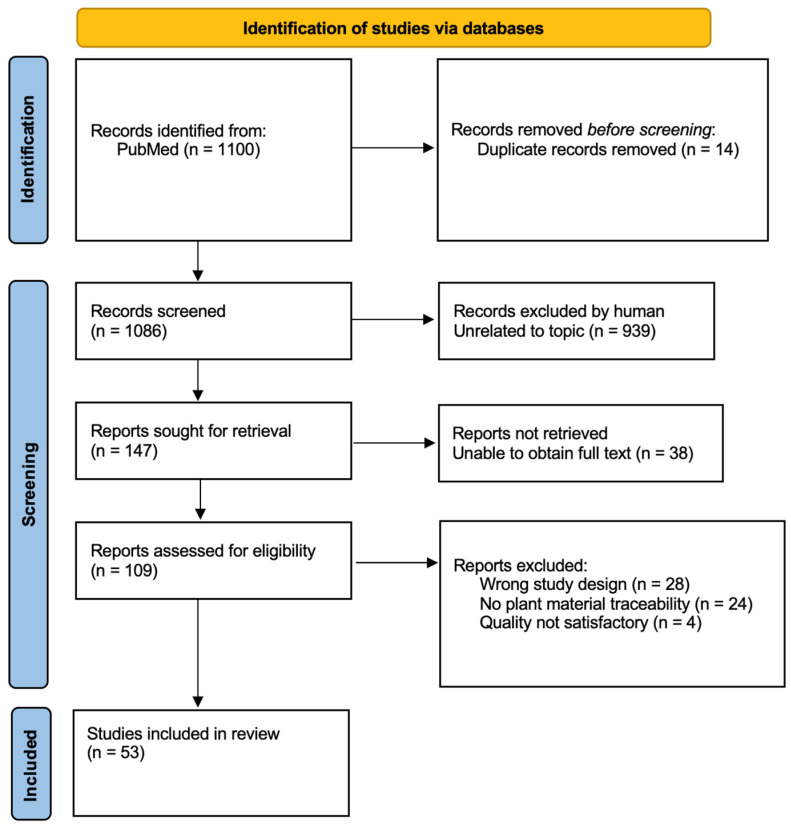
PRISMA diagram of the detailed study selection process.

Following the initial literature search, duplicates (n = 14) were removed automatically by Rayyan, and 1086 articles were included in the initial screening process, which consisted of title screening for relevancy to the topic. After the title screening, 939 articles were excluded because they were unrelated to medicinal uses. Next, 147 reports were sought out for full-text retrieval, and a handful of them were not retrieved (n = 38); therefore, they were excluded from this review. Full-text screening was performed on 109 articles, and after rigorous screening of the data presented and applying the inclusion criteria, we established a final number of 53 articles to be included in the study. Thus, from the articles considered relevant to the topic of the study, 48.6% also met the inclusion criteria. The screening process is presented in more detail in Figure 1.

### 3.2. Studies Overview

The included studies were analysed rigorously, and relevant data regarding therapeutic and medicinal uses were extracted from them. A general overview of all the included studies can be found in Table 1. The studies that passed the criteria evaluation and quality assessment for each studied plant are as follows: 38 studies on *Ailanthus altissima*, 6 studies on *Amorpha fruticosa*, and 9 studies on *Phytolacca americana*. Although the plant *Asclepias syriaca* was included in the initial literature search, none of the retrieved articles met the evaluation criteria because they were either irrelevant to the topic or did not report at least one type of activity assay that could be correlated to a therapeutic or medicinal use. Considering that understanding the potential use in these fields constituted the main objective of this study, the identified studies that did not meet this criterion were excluded. Among them, we have identified and included studies focusing on testing the properties of both crude extracts and isolated compounds, either obtained directly from the plant through isolation or by purchasing and directly testing the purified compound of the selected plant origin.

**Figure 2:**
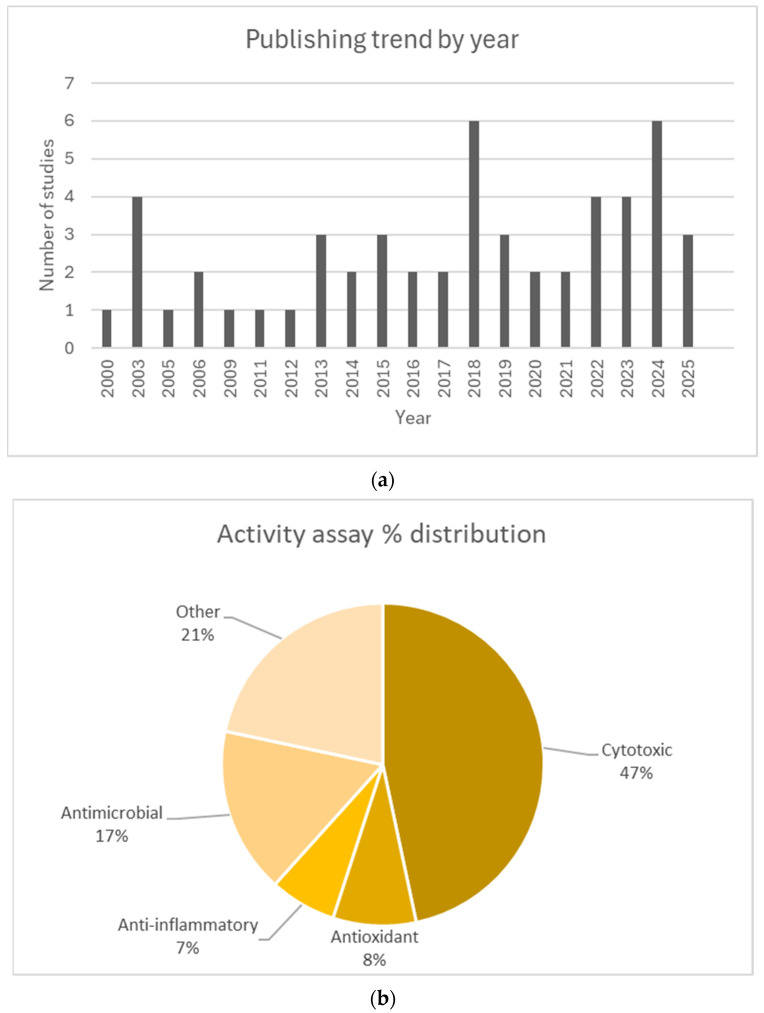
(**a**) Publishing trends of included studies by year. (**b**) Distribution of activity assays for included studies.

Data analysis was conducted to identify patterns and trends in publishing, as well as the distribution of biological activities that were assessed, as found in Figure 2. Most of the studies included, i.e., 47%, evaluated the cytotoxic potential of the selected INPS and of some isolated compounds obtained from them on various cell lines to serve as preliminary research for potentially developing new antitumor agents. The antimicrobial activity, including antibacterial and antiviral assays, is also a frequent topic of research among the selected studies (17% of the total studies), followed by antioxidant activity (8% of the total studies) and anti-inflammatory activity (7% of the total studies). Amongst other tested activities, we noticed more specific types of activities, such as various enzyme inhibition assays, neuroprotective effect assays, DNA protective assays, and cardiovascular effect assays, to name a few.

In the following sections, each study will be discussed in more detail with a focus on the therapeutic/medicinal use analysed, the potential applications in the medical field, the limitations and gaps identified, and the future directions of research for these specific topics.

### 3.3. INPS and Their Antimicrobial Potential

#### 3.3.1. The Antibacterial Activity of INPS

There is no doubt that the search for new antibacterial agents is an essential and currently ongoing topic of research in the medical and pharmaceutical fields. Bacterial resistance to the conventionally used antibiotics is on the rise due to the high adaptability of bacteria and their overall evolutionary nature, the inappropriate prescribing and use of antibiotics (overuse and misuse), environmental contaminations, the use of large-spectrum antibiotics, and the under-utilisation of antibiograms in medical practice [90,91,92,93].

Plants have been used historically for their antibacterial properties [94], and screening the phytocomponents of plants is one of the strategies for discovering new therapeutic agents that could help combat the crisis of bacterial resistance [95,96,97]. For the chosen plants, we have identified a range of tested bacterial strains, including a clinical isolate. The growth inhibition activity was tested on crude extracts, chromatography-fractionated extracts from various plant parts, and on some isolated compounds. The findings are summarised in Table 2.

The most predominant plant material screened among the selected studies is the aerial part of *Ailanthus altissima* (bark and leaves). It was the only plant with studies assessing how the phytochemical composition and the activity can differ in material collected from different locations [70] or collected during different seasons and processed differently before the extraction [73]. Other INPS could also benefit from similar studies of the variation of the phytochemical content and the biological activity depending on the location, time of harvest, and pre-extraction processing techniques. Such studies would lead to a better understanding of the best time for harvesting to maximise their value and applicability, depending on each specific geographic location.

In terms of the bacteria and methods selected in the presented studies, the range varied greatly. A study conducted by Patra JK et al. assessed the bacterial growth inhibition effect of *Phytolacca americana* crude extracts and some of their fractions on two bacteria involved in periodontal diseases and the formation of oral caries, with the main findings being that they were highly active on *P. gingivalis* and moderately active on *S. mutans*. Therefore, it was concluded that the extract could be formulated in the form of oral hygiene products or drugs for oral diseases [87]. However, the study used no antibiotic control as a reference except for the solvent and inoculum controls. Although the bactericidal effect was evaluated, only the minimum inhibitory concentrations and growth inhibition percentages were determined. Future studies could benefit from including the determination of the minimum bactericidal concentrations, the use of antibiotic controls, and in vivo determinations.

Another interesting approach for determining antibacterial activity, aside from the conventional micro-dilution method that is most often employed, was used by Kim YS et al., where rotenoids and flavanones isolated from the root of *Amorpha fruticosa* were assessed for their bacterial neuraminidase inhibition capabilities. Among the isolated compounds, amorisin was 30 times more potent in inhibiting bacterial neuraminidase originating from *Clostridium perfringens* than the quercetin control, an already established neuraminidase inhibitor. The promising results led to determinations of the inhibition of biofilm production on live bacteria (*Pseudomonas aeruginosa*), in which two of the compounds isolated, isoamoritin and dalbinol, showed the best efficiency [76].

Generally, the selected studies focused on testing some common bacterial strains, both Gram-positive and Gram-negative, used in antibacterial testing and utilised the micro-dilution assay method on microplates to help determine the MIC of the tested plant extracts or isolated compounds. The limitations of these studies include the fact that they took place in the laboratory under controlled conditions. In vivo studies should also be employed to determine their activity with biological variability in mind, and studies should be performed to evaluate the mechanism of actions, pharmacokinetics, and toxicology for the most promising agents discovered. The real applicability of the results should also be assessed by comparing the results to standard antibiotic controls, where relevant.

Interestingly, some studies showcase a synergistic effect of plant extracts, particularly those rich in phenolic compounds, with conventional antibiotics, which have proved to help combat various bacterial infections, as they can potentiate the effect of antibiotics and even combat antibacterial resistance [98,99,100]. This presents another valuable research opportunity for the selected plants, given the lack of published data on this topic.

#### 3.3.2. The Antiviral Activity of INPS

Viral infections are a significant cause of concern globally, with many causing annual epidemics that result in a loss of lives. Managing epidemics and pandemics is a challenging task; therefore, there is a constant need to find new antiviral agents, especially since viruses may adapt to classic antiviral drugs [101,102,103,104]. As with antibacterial agents, screening plants and isolating phytochemicals from them is a good strategy for discovering and developing new antiviral agents [105].

From the identified studies assessing the antiviral activity of the selected plants, the majority of the them were focused on testing the effect of pokeweed antiviral protein (PAP) on inhibiting the growth of a few viruses, such as Japanese encephalitis virus (JEV), human immunodeficiency virus 1 (HIV-1), human T-cell leukaemia virus I (HTLV-I), and Epstein–Barr virus (EBV). PAP is a plant-derived N-glycosidase ribosomal-inactivating protein, and it is isolated from the plant *Phytolacca americana*. Once purified from the plant, PAP can also be obtained through cloning, which is useful in laboratory settings to produce larger quantities for research purposes. *Ailanthus altissima* stem bark crude methanolic extract was also screened for its potential effect against HIV-1, and some isolated compounds from the aerial parts of the same plant were screened against the Epstein–Barr virus. The main results for each study are summarised in Table 3.

Aside from the growth inhibition effects, Ishag HZ also noted a prophylactic effect of PAP when it was tested in vivo against JEV infection. When PAP was administered in mice before the injection of a lethal dose of JEV, a survival rate of 87.50% was obtained, which was higher than the survival rate when PAP was administered post-infection (85.70%). PAP also dose-dependently inhibited JEV replication in vitro with an IC_50_ of 23.10 nM. PAP also showed inhibitory activity on both HIV-1 and HTLV-1 [82]. Zhabokritsky A et al. performed a mechanistic study on the effect of PAP on HIV-1, and the authors determined through Western blotting that the protein can reduce HIV-1 production by altering the splicing of viral mRNA through targeting Rev, a regulatory protein of HIV-1. Therefore, the authors concluded that PAP should be further studied for its antiviral activity against HIV-1 [84].

Another selected study by Chang YS et al. determined that the *Ailanthus altissima* stem bark methanolic extract was able to inhibit HIV-1 fusion by 74.90% at a dose of 100.00 μg/mL, concluding that there is a need to isolate the active components from the tested extract to find potential new anti-HIV candidates [52]. Isolated compounds from the aerial parts of *Ailanthus altissima*, namely the quassinoids ailantinol E, F, and G, were studied by Tamura S et al. The authors determined that they exhibited an inhibitory effect on Epstein–Barr virus early antigen activation in Raji cells (in vitro), which may prevent EBV-associated cancers [50]. Similarly, antiviral activity combined with possible cancer prevention effects was also determined by Mansouri et al., who found that PAP diminished virus production of HTLV-1 by affecting it at both the translational and transcriptional levels [83].

The antiviral potential can be further explored by isolating additional compounds from promising extracts and testing their antiviral activity. Furthermore, employing in vivo, mechanism of action, pharmacokinetic, and clinical studies can help develop compounds with potential as novel antiviral agents.

#### 3.3.3. The Antimalarial Activity of INPS

In the screening process carried out, we found one study that assessed the in vitro antimalarial activity of both the ethanolic seeds and seedlings extracts, as well as the fractions and isolated compounds, of two quassinoids (ailanthone and 6α-tigloyloxychaparrinone) from *Ailanthus altissima*. The authors chose a chloroquine-sensitive strain (HB-3) and a chloroquine-resistant strain (Dd-2) of *Plasmodium falciparum*. In addition to the antimalarial assay, the authors also performed a cytotoxic in vitro assay. Both ailanthone and 6α-tigloyloxychaparrinone exhibited antiplasmodial activity against sensitive and resistant *Plasmodium falciparum* strains, with ailanthone yielding superior results (0.003 μg/mL against HB-3 and 0.037 μg/mL against Dd-2) compared to those of 6α-tigloyloxychaparrinone (0.061 μg/mL against HB-3 and 0.062 μg/mL against Dd-2). However, in the cytotoxic assay on the Vero cell line, ailanthone showed a low cytotoxicity at 200.00 μg/mL, whereas 6α-tigloyloxychaparrinone showed no toxicity at the same concentration [51]. Additional studies, both in vitro and in vivo, should be performed to assess the potential of ailanthone and 6α-tigloyloxychaparrinone as antimalarial/antiplasmodial agents. Unfortunately, the evidence of antimalarial activity is limited when it comes to *Ailanthus altissima*, considering that the one selected study did not describe a mechanism of action for the two quassinoids, nor did it compare the results to standard antiplasmodial drugs. Moreover, there were no in vivo, bioavailability, and toxicity studies performed, which would otherwise provide more clues into the safety profile and actual applicability of the in vitro results in clinical settings.

### 3.4. INPS and Their Antioxidant Potential

Oxidative stress is caused by an imbalance of pro-oxidant and antioxidant molecules, which results in disruptions of the redox balance in the organism and cell damage. The human organism is equipped with endogenous antioxidant enzymes that can deal with oxidative stress, such as glutathione peroxidases (GSH-Px), superoxide dismutases (SOD1 and SOD2), and catalases (CAT). Even though pro-oxidant molecules are needed, to some degree, for normal body function, specifically in redox processes, in some cases, excess reactive oxygen species (ROS) production can overwhelm the organism and its endogenous enzymes, leading to cell damage [106]. In such cases, supplementing the organism with antioxidant compounds that can assist in capturing free radicals generated by ROS can help prevent cell damage. An excess amount of ROS and oxidative damage to cells has been linked to various pathologies such as cancer, cardiovascular diseases, and diabetes, to name a few [107]. Plants are naturally equipped with many phytocomponents that can deal with oxidative stress, as this is their way of adapting to the environmental stress factors (UV rays, predators, heat, cold, drought, flooding, soil composition, etc.), thus making them good candidates for the extraction of antioxidant compounds [108,109].

In terms of the assays used for determining the antioxidant capacity of various extracts and compounds isolated from the selected plants, most of which are found in the selected studies, in vitro methods that use generated free radicals that are then partially captured by the antioxidant molecules were commonly employed. However, these assays do not generally correlate well with in vivo results because they cannot predict the antioxidant effect in the context of the complex biological processes in the human body and the capacities of the endogenous enzymes to deal with pro-oxidant molecules; this may lead to potential overestimation of the results [110,111]. Consequently, in vitro assays remain the initial step in determining the antioxidant potential of compounds/extracts to identify the most promising types. Thus, to more accurately predict the significance of the results and their antioxidant potential in therapy, in vivo tests are required. Unfortunately, most of the selected studies fall short in this regard; thus, they can be considered only preliminary results. The main findings regarding antioxidant potential can be found in Table 4.

Among the selected studies, few assessed the in vivo antioxidant activity. Popovici LF et al. performed a comprehensive in vivo study on the anxiolytic, neuroprotective, and antioxidant effects of *Phytolacca americana* hydroethanolic fruit extracts on scopolamine-treated zebrafish. The authors found that the extracts showed the ability to enhance the enzymatic activity of the endogenous SOD, CAT, and GSH-Px, while at the same time reducing lipid and protein oxidation markers. The results were linked to a neuroprotective effect, considering the design of the study and the fact that oxidative stress is associated with neurodegenerative diseases [89]. A similar approach was employed by Rahman HMA et al., in which scopolamine-induced male Sprague Dawley rats were treated with *Ailanthus altissima* methanolic bark extracts in doses of 50.00, 100.00, and 200.00 mg/kg for 28 days. The antioxidant activity of the extracts was then determined on isolated brains obtained from the studied specimens. The results showed a significant dose-dependent increase in the activity of both SOD and GSH-Px enzymes in the groups treated with *Ailanthus altissima* bark extract compared to the results for the controls. Moreover, malondialdehyde (MDA), a marker of lipid peroxidation and oxidative stress, was significantly reduced in brains treated with bark extracts compared to the levels in the scopolamine controls [63]. Once again, these results showcased an in vivo antioxidant capacity of the extract that can be correlated to a subsequent neuroprotective effect.

Most of the studies focus on the in vitro antioxidant activity of the selected plants, which constitutes a limitation. More studies aiming to assess in vivo antioxidant activities should be performed to demonstrate the potential therapeutic application in the biological context, starting with mechanism of action studies and animal studies and moving on to clinical trials, if the initial results are promising. However, it is necessary to continue preliminary in vitro screening of the antioxidant potential of plants.

### 3.5. INPS and Their Cytotoxic Potential

**Figure 3:**
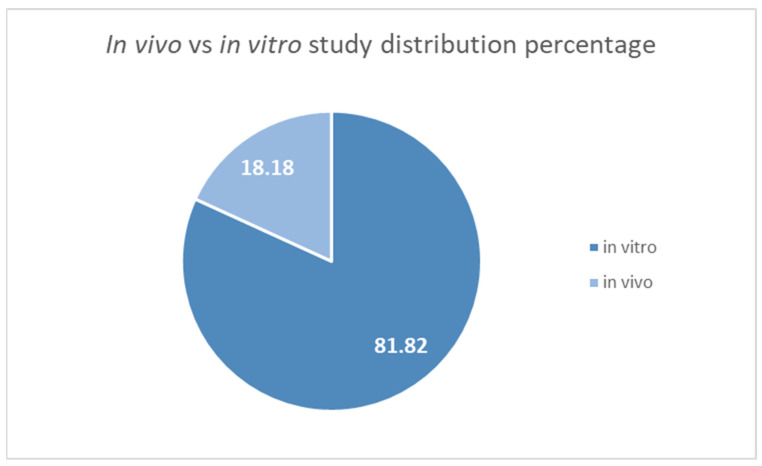
Distribution percentage of in vivo vs. in vitro assays performed in cytotoxic studies.

Plant extracts contain various components that exhibit cytotoxic activity, generally depending on their concentration [112,113,114]. Cytotoxicity comprises two critical aspects; one is a toxicological aspect, in which extracts and contained compounds can be assessed for their capacity to damage normal human cells at specific concentrations, and this is used to determine their safety profile. The other aspect is assessing their cytotoxicity on tumoral cells at various concentrations, with potential applications and benefits in cancer therapy, adjuvants, and alternative treatments. It is essential to determine both aspect to ensure a good safety profile for administration, but also for ensuring effectiveness against tumoral cells. Plant extracts and compounds may exhibit specificity for different tumour cells and pathological markers, and lower specificity and toxicity on normal human cells, which we will discuss in the following paragraphs. As with all the other assessed activities, it is just as essential to correlate in vitro assays with in vivo ones, and if deemed safe and effective, clinical trials may be performed [115,116,117]. Among the selected studies, the distribution of in vitro and in vivo cytotoxicity studies is visualised in Figure 3, highlighting the need for more in vivo studies. Furthermore, the studies that reported cytotoxic activity among the selected plants is summarised in Table 5.

Ailanthone isolated from *Ailanthus altissima* (Table 5) is most often studied in the context of cytotoxic antiproliferative activities among the selected plants. We also identified a few more studies that further observe its activities and elucidate its mechanisms of action. Cucci MA et al. determined that ailanthone is capable of reducing the proliferation and invasion rates of A2780/CP70 ovarian cancer cell lines that are resistant to cisplatin (CDDP) treatment in vitro through a varied mechanism of action, specifically by inhibiting Nrf2 (nuclear factor erythroid 2-related factor 2) and YAP/TEAD (yes-associated protein 1 and the transcription factor) transcriptional activity and by reducing UCHL1 (ubiquitin carboxy-terminal hydrolase L1) expression, which both lead to increased oxidative stress in the cancerous cells [41]. Similarly to Cucci MA et al., Daga M et al. determined the in vitro activity of ailanthone on bladder cancer cell lines that are resistant to CDDP treatment. They observed that it was able to inhibit the proliferation and invasion of the cancerous cell lines by reducing the expression levels of the Nrf2, YAP, and c/Myc (an oncogene). Moreover, the authors determined that ailanthone showed a low cytotoxic profile on normal kidney cells, highlighting a specific selectivity of the compound for cancerous cells [40]. Both these studies could serve as a basis for future in vivo studies in this context, since they highlight ailanthone’s potential in treating tumours that are resistant to conventional treatment.

Another compound of interest that was isolated from *Ailanthus altissima* was 1-methoxy-canthin-6-one, with some of its activities presented in Table 5. In addition, a study conducted by Ammirante M et al. also highlighted its apoptotic properties on a handful of cancerous cell lines, namely on the Jurkat cell line (leukaemia), the NPA and ARO cell lines (thyroid carcinoma), and the Huh7 cell line (hepatocellular carcinoma). For all tested cell lines, apoptosis was evident at a concentration of under 10 µmol/L, with the IC_50_ being approximately 40 µmol/L. The authors also noted that apoptosis occurred through the MAPK (mitogen-activated protein kinase) pathway, since 1-methoxy-canthin-6-one modulated JNK (c-Jun NH_2_-terminal kinase), while at the same time synergising with the hrTRAIL (human recombinant TNF-related apoptosis-inducing ligand) [55]. Thus, 1-methoxy-canthin-6-one might also be potentially helpful in anticancer therapy.

Furthermore, we identified six studies assessing cytotoxic activity in vivo, all of them being focused on ailanthone isolated from *Ailanthus altissima*. Fang C et al. determined that ailanthone was able to effectively inhibit NSLCL subcutaneous tumour growth and metastasis in tumour-bearing mice at doses of 0.10 mg/kg, 1.00 mg/kg, or 2.00 mg/kg, with the best result at a dose of 2.00 mg/kg. Treatment with ailanthone also enhanced the survival rate of the tested specimens. Moreover, the authors found that the antitumour mechanism of ailanthone was based on modulating the UPF1/GAS5/ULK1 (up-frameshift protein 1/growth arrest specific 5/unc51-like autophagy activating kinase 1) signalling pathway [46]. Liang J et al. studied the effects of ailanthone on lung metastasis associated with osteosarcoma. Here, the authors found that the compound was able to suppress metastasis by targeting the KMT2A-MEN1 gene complex and inhibiting the SSP (serine synthesis pathway) in vivo in mouse models, without exhibiting significant toxicity on normal cells. Also, the authors noted that ailanthone exhibited a different mechanism pathway compared to that of conventional osteosarcoma therapeutic agents [48]. Similarly, Zhang Y et al. studied the effect of ailanthone on osteosarcoma, finding that when administered, it significantly inhibited tumour growth in Saos-2 mice xenografts, without causing significant weight difference between the ailanthone-treated lot and the DMSO (dimethyl sulfoxide) control lot, thus displaying good tolerability. The authors also showed that the SSP pathway was most significantly modulated by ailanthone, which was also one of the findings in the study conducted by Liang J et al. [44,48]. Li J et al. studied the in vivo cytotoxic activity of ailanthone on subcutaneous bladder cancer mouse models. They determined that treatment with the compound resulted in reduced tumour growth by modulating the JAK/STAT3 (Janus kinases/signal transducer and activator of transcription 3) pathway, suppressing its activity [49]. Wang Y et al. sought to determine the cytotoxic activity of ailanthone in the context of breast cancer-related bone metastasis, finding that ailanthone significantly suppressed the growth of bone metastasis cells in the tested mouse models when compared to the results for the control group. Together with data from in vitro studies, the authors concluded that ailanthone was capable of blocking osteoclast differentiation in bone metastasis by inhibiting cytokines that are secreted by the breast cancer cells, mainly through the RANKL (receptor activator of nuclear factor kappa B ligand)-dependent pathway [45]. Finally, the last in vivo cytotoxic activity assessment that we identified among the selected studies was conducted by Zhuo Z et al., in which ailanthone was tested for its effects against hepatocellular carcinoma. The study performed on tumour xenografts in nude mice showed that ailanthone was capable of significantly inhibiting tumour growth compared to the results for the control group, while also maintaining a good safety profile. The authors also observed that ailanthone increased the apoptosis marker TUNEL (terminal deoxynucleotidyl transferase (TdT) dUTP nick-end labelling) in vivo and that it induced G_0_/G_1_-phase cell cycle arrest, observed through the significantly lower expression levels of the key regulatory protein cyclin-dependent kinase 4 (CDK4) in the treated group compared to those in the control group [37].

Crude extracts were not often studied in this antiproliferative context. However, a few data points about their cytotoxicity are tabulated. In addition, we identified a study by Cui X et al., which focused on determining the antiproliferative activity of the 90% crude ethanolic extract obtained from *Amorpha fruticosa* leaves on HCT116 and HepG2 cell lines. The authors reported a cell viability percentage of under 60% for the extract concentrations of 100.00 µg/mL, 25.00 µg/mL, and 5.00 µg/mL on the HCT116 cells; however, no activity was observed on HepG2 cells [78].

Concerning the *Phytolacca americana* plant, one study conducted by Saleri FD et al. showed a possible anticancer activity of the crude ethanolic extract on two cell lines, namely SCG-7901 and Hep G2; however, the study was designed as a comparative assessment with other *Phytolacca* species. As such, it was concluded that in this specific context, *Phytolacca acinosa* exhibited greater antiproliferative activity (IC_50_ = 27.20 ± 1.60 µg/mL for the SCG-7901 cell line and IC_50_ = 25.59 ± 1.63 µg/mL for the Hep G2 cell line) when compared to that of *Phytolacca americana*, for which the authors provided no conclusive values [88].

Notably, plant-derived compounds display certain limitations in cancer therapy, mainly, as discussed previously, the issue of promising in vitro results that do not translate well into real-life settings and in actual clinical practice. There are a few factors, such as limited bioavailability, poor solubility, high toxicity, incompletely deciphered mechanisms of action, and the possibility of drug resistance development, that limit this translation. Therefore, more research is needed in this area, including the preliminary screening of promising compounds, complete elucidation of the mechanisms of action, identifying target molecules/pathways, determining safety profiles, and establishing the actual applicability of potential novel therapeutic agents in clinical settings.

### 3.6. INPS and Their Other Therapeutic Potentials

Among the selected studies, we have identified even more therapeutic applications of broad applicability; however, there is a need for more studies in these areas to properly assess the therapeutic potential in clinical practice. The possible therapeutic applications identified ranged from enzyme inhibition, which was focused on acetylcholinesterase (AChE), butyrylcholinesterase (BuChE), and tyrosinase inhibition, as well as anti-inflammatory, antidiabetic, neuroprotective, neurotrophic, and DNA protective potentials and potential application in the cardiovascular system. Data concerning enzyme inhibition, anti-inflammatory, and neuroprotective activities are summarised in Table 6.

Weidner C et al. and Lee W et al. both tested isolated compounds from *Amorpha fruticosa* for their antidiabetic activity [75,79]. Weidner C et al. focused on amorfrutins and determined that they were capable of selectively binding and activating PPARγ (peroxisome proliferator-activated) receptors, and when amorfrutin 1 was administered to C57BL/6 mice with high-fat diet-induced obesity, it was able to reduce insulin resistance, with an effect equal to that of rosiglitazone (used as a control). Amorfrutin 1 also enhanced glucose tolerance, increased insulin sensitivity, and lowered plasma levels of triglycerides, free fatty acids, insulin, and glucose. All the results were comparable to those for rosiglitazone, with the absence of a hepatotoxic effect. Moreover, treatment with amorfrutin 1 (3 weeks) also significantly reduced body weight gain in the tested mice when compared to the results for the rosiglitazone control. When amorfrutin 1 was tested against *db*/*db* mice with a leptin-receptor deficiency, a genetic model for type II diabetes, the results showed a more substantial reduction in insulin concentrations in the plasma compared to those for rosiglitazone. Moreover, its administration had no significant effect on body weight gain compared to that for the rosiglitazone-treated mice, who displayed an increase of 30% in body weight after administration. Similar to the levels for the diet-induced obesity mouse models, amorfrutin 1 also lowered plasma triglycerides, fatty acids, and glucose in *db*/*db* mice and enhanced insulin sensitivity. The authors noted a preservation of the pancreatic function in *db*/*db* mice treated with amorfrutin 1 as well, which was connected to improvements in insulin levels in the treated group compared to those in the control [75]. Lee W et al. focused on a different compound isolated from the fruits of *Amorpha fruticosa*, namely 5,7-dihydroxy-6-geranylflavanone (DGF). Through in vitro determinations, the authors found that DGF binds and activates PPARα and PPARγ receptors, promoting fatty acid oxidation (through activation of PPARγ receptors), improving insulin sensitivity, and enhancing glucose uptake mediated by the IR-Akt (insulin receptor- protein kinase B) signalling axis. No in vivo experiments were performed in this study [79].

Next, we have identified some studies focusing on neurological applications, ranging from neuroprotective to neurotrophic effects, as well as anxiolytic effects of certain extracts or compounds obtained from the selected plants. Some of these can be found in Table 6 for the AChE and BuChE in vitro inhibition assays. The inhibition of both enzymes has been linked to a neuroprotective effect and therefore, to the prevention of neurodegenerative diseases, although in vivo tests should also be employed to assess the actual applicability of these results [118,119,120]. Some other studies related to neurological applications are discussed in the following paragraphs.

A study conducted by Kim SR et al. focused on determining both the in vitro and in vivo anti-inflammatory effects of the ethanolic *Ailanthus altissima* leaf extract on LPS (lipopolysaccharide)-induced primary astrocytes and on LPS-injected mice brains in a neurological context. During the in vitro studies, the authors found that the extract was able to suppress inducible nitric oxide synthase (iNOS) production in LPS-induced astrocytes, thus inhibiting nitric oxide (NO) release; inhibit the upregulation of TNF-α, IL-1β, and IL-6 expression; inhibit ROS production; inhibit NF-κB (nuclear factor kappa B) translocation, which in turn suppressed proinflammatory cytokines; and inhibit the activation of the MAPK pathway through suppressing the activation of ERK (extracellular signal-regulated kinase) and JNK (c-Jun NH2-terminal kinase). In vivo neuroinflammation studies were performed on LPS-injected mice who were subjected to the Y-maze and three-chamber tests after being treated with the extract. The authors concluded that the extract was able to lower LPS-induced memory and social impairments in mice, and further Western blot studies confirmed some of the effects of the extract that were observed in the in vitro studies, namely that in the LPS-injected brain tissue of extract-treated mice, the iNOS and COX-2 expression was suppressed, and the MAPK pathway activation was inhibited. Thus, the authors concluded that *Ailanthus altissima* may have potential applications in the treatment of diseases associated with neuroinflammation [67].

Another interesting approach in the neurological area was published by Takahasi et al., who performed an in vitro neurotrophic study on cortical neurons from rats using 1,4-benzodioxane derivatives isolated from the seeds of *Phytolacca americana*. Among the tested compounds, americanoic acid A methyl ester exhibited the most potent neurite outgrowth promotion activity, and americanin-type four also induced neuritogenesis. Thus, the authors concluded that with more evidence, these compounds could potentially be considered as neurotrophic agents [85].

Next, we have identified two in vivo neuroprotective studies among those selected. First off, Popovici LF et al. studied the effects of *Phytolacca americana* fruit extract on a scopolamine-induced memory-impaired zebrafish (*Danio rerio*) model by performing various in vivo behavioural assays and an AChE activity determination on brain samples collected from the tested specimens. After the assays were performed, the authors concluded that the fruit extracts exhibited an anxiolytic effect, as shown by the lowering of the anxiogenic impact of scopolamine and improved scopolamine-induced cognitive impairment during behavioural tasks. Coupled with the data obtained from determinations on the zebrafish brain that showed a reduction of brain oxidative stress and the inhibition of AChE activity, the authors concluded that *Phytolacca americana* fruit extracts could potentially be used in treating anxiety and cognitive impairment associated with amnesia [89]. Muhammad Abdur Rahman H et al. performed behavioural studies similar to those of Popovici LF et al., using bark extracts from *Ailanthus altissima* on Sprague Dawley rats. They used scopolamine to induce amnesia and cognitive impairment, as needed. The results of the behavioural experiments showed an anxiolytic effect and improvement of memory in the treated group compared to the results for the control. Moreover, the analysis of brain isolates from the tested specimens showed an inhibition of the activity of both MDA and AChE, which may explain the effects noticed in the behavioural tests [69].

We also identified one study that focused on some potential therapeutic applications for the cardiovascular system. Rahman HMA et al. aimed to assess the hypotensive, smooth muscle relaxant, and anticoagulant effects of Ailanthus altissima bark extracts by employing specific assays in vitro on isolated organs from rabbits, Sprague Dawley rats, and BALB/c mice. The hypotensive effect was also tested in vivo on rats. Based on the results, the authors concluded that the extracts might possess calcium channel blocking compounds. However, further studies should be conducted to isolate the specific compounds responsible for this effect, to determine their exact mechanisms, and to perform more in vivo pharmacological and toxicological studies to assess their true potential in this context [63].

Finally, Andonova T et al. determined the in vitro DNA protective effect of several extracts from *Ailanthus altissima*, finding that flower extracts displayed the most promising results in the DNA nicking in vitro assay [71]. Similar to other identified potential applications, more studies should be undertaken to properly assess the potential of this plant in DNA protection. More specifically, the compounds that are responsible for this effect could be isolated, and mechanistic studies could be conducted on them. Further studies could consist of safety profiles, in vivo testing, and clinical studies to understand the true potential of the DNA protective effect.

## 4. Study Limitations

The present review has several limitations that are highlighted further:

- Most of the included research is based on in vitro assays, which may not accurately reflect therapeutic efficacy in vivo;
- The exclusion of non-English publications and reliance on a single database (PubMed) may have led to the omission of relevant studies.
- The lack of standardised methodologies across the reviewed articles limits direct comparability of results;
- The absence of toxicological and pharmacokinetic data for many compounds restricts the assessment of their clinical applicability.

Thus, the study provides a valuable foundation for future research and highlights the biomedical potential of INPS.

## 5. Conclusions

The world of plants has proved to be a valuable source of bioactive compounds with various applications in therapy, as they offer alternatives to conventional treatments. The spread of INPS can have adverse effects on the environment and society; therefore, valorising their biomass through therapeutic applications presents a dual benefit: ecological management and biomedical innovation. To achieve this valorisation, we must understand what is currently known about INPS in this context and determine the gaps and future directions. Our review has focused on four selected INPS, but a clear prioritisation of species and compounds based on therapeutic promise is still needed. For example, ailanthone from *Ailanthus altissima* and amorfrutins from *Amorpha fruticosa* emerged as up-and-coming candidates, yet their clinical relevance remains underexplored.

As this present study’s findings suggest, *Ailanthus altissima* and its isolated compound ailanthone have been the topic of most of our selected studies, which focused on the antiproliferative activity, anti-inflammatory properties, and antimicrobial potentials. *Amorpha fruticosa* has potential uses in managing metabolic diseases such as type II diabetes, while also exhibiting antiproliferative and neuroprotective effects. *Phytolacca americana* may find potential uses in pathologies related to increased oxidative stress, neurological diseases, and in formulating novel antiviral agents through its pokeweed antiviral protein. Finally, due to the set eligibility criteria, none of the retrieved papers on the topic of *Asclepias syriaca* were suitable for inclusion.

In summary, we acknowledge that all the selected INPS contain potentially valuable bioactive compounds that can exert various therapeutic effects that may be useful in the medical and pharmaceutical fields. However, more comprehensive studies are needed to properly assess their biological potential, from complete screenings of the entire plant material harvested from various geographical regions to preliminary in vitro assessments that should be completed by in vivo determinations to further broaden the knowledge and understanding of how the plant material can be best utilized in the context of therapeutical applications in the medical and pharmaceutical fields. For those compounds and/or extracts that show the most potential, pre-clinical and clinical studies should be employed to determine their actual applicability.

## Figures and Tables

**Table 1:** Summary of included original research articles by plant species, biological activity, origin of plant material or compounds, and non-native status.

Author, Year	Plant/Compound	Activity	Origin	Non-Native (Yes/No)	Reference
Zhuo Z et al., 2015	Ailanthone	Cytotoxic	Jinan University, Guangzhou, China	N/A	[37]
Wei C et al., 2018	Ailanthone	Cytotoxic	Jinan University, Guangzhou, China	N/A	[38]
Han F et al., 2018	Ailanthone	Cytotoxic	Puruifa Science & Technology Development Co., Chengdu, China	N/A	[39]
Daga M et al., 2019	Ailanthone	Cytotoxic	Baoji Herbest, Bio-Tech Co., Ltd., Baoji, China	N/A	[40]
Cucci MA et al., 2020	Ailanthone	Cytotoxic	Baoji Herbest, Bio-Tech Co., Baoji, China	N/A	[41]
Ding H et al., 2021	Ailanthone	Cytotoxic	Shanghai Yiyan Biotechnology Co., Ltd., Shanghai, China	N/A	[42]
Wang S et al., 2022	Ailanthone	Cytotoxic	Jiangxi Herb Tiangong Technology, Nanchang, China	N/A	[43]
Zhang Y et al., 2022	Ailanthone	Cytotoxic	GlpBio Technology, Montclair, CA, USA	N/A	[44]
Wang Y et al., 2023	Ailanthone	Cytotoxic	MedChemExpress Ltd., Monmouth Junction, NJ, USA	N/A	[45]
Fang C et al., 2024	Ailanthone	Cytotoxic	Dasfbio Nanjing, Nanjing, China	N/A	[46]
Yang H et al., 2024	Ailanthone	Cytotoxic	Chengdu Alfa Biological Technology Co., Ltd., Chengdu, China	N/A	[47]
Liang J et al., 2025	Ailanthone	Cytotoxic	GlpBio Technology, Montclair, CA, USA	N/A	[48]
Li J et al., 2025	Ailanthone	Cytotoxic	ChemFaces, Wuhan, China	N/A	[49]
Tamura S et al., 2003	*Ailanthus altissima*	Antiviral	Taiwan	No	[50]
Okunade AL et al., 2003	*Ailanthus altissima*	Antimalarial	United States	Yes	[51]
Chang Y et al., 2003	*Ailanthus altissima*	Antiviral	South Korea	Yes	[52]
De Feo V et al., 2005	*Ailanthus altissima*	Cytotoxic	Italy	Yes	[53]
Jin MH et al., 2006	*Ailanthus altissima*	Anti-inflammatory	South Korea	Yes	[54]
Ammirante M et al., 2006	*Ailanthus altissima*	Cytotoxic	Italy	Yes	[55]
Wang Y et al., 2013	*Ailanthus altissima*	Cytotoxic	China	No	[56]
Kim HM et al., 2015	*Ailanthus altissima*	Anti-inflammatory	South Korea	Yes	[57]
Kim H et al., 2016	*Ailanthus altissima*	Anti-inflammatory	South Korea	Yes	[58]
He Q et al., 2018	*Ailanthus altissima*	Cytotoxic	China	No	[59]
Wang R et al., 2018	*Ailanthus altissima*	Cytotoxic	China	No	[60]
Yan ZY et al., 2018	*Ailanthus altissima*	Cytotoxic	China	No	[61]
Jeong M et al., 2018	*Ailanthus altissima*	Cytotoxic	South Korea	Yes	[62]
Rahman HMA et al., 2019	*Ailanthus altissima*	Hypotensive Anticoagulant Smooth muscle relaxant	Pakistan	Yes	[63]
Du YQ et al., 2019	*Ailanthus altissima*	Neuroprotective	China	No	[64]
Yan ZY et al., 2020	*Ailanthus altissima*	Cytotoxic	China	No	[65]
Mo Y et al., 2021	*Ailanthus altissima*	Antioxidant	Bozhou Baohua Pharmaceutical Co., Ltd. Bozhou, China	N/A	[66]
Kim SR et al., 2022	*Ailanthus altissima*	Anti-inflammatory	South Korea	Yes	[67]
Gao ZH et al., 2022	*Ailanthus altissima*	Cytotoxic	China	No	[68]
Muhammad Abdur Rahman H et al., 2023	*Ailanthus altissima*	Neuroprotective Antioxidant Anti-enzymatic	Pakistan	Yes	[69]
Boukhibar H et al., 2023	*Ailanthus altissima*	Antibacterial	Algeria Tunisia	Yes	[70]
Andonova T et al., 2023	*Ailanthus altissima*	Antioxidant DNA protective	Bulgaria	Yes	[71]
Song Q et al., 2024	*Ailanthus altissima*	Anti-enzymatic	China	No	[72]
Cocîrlea MD et al., 2024	*Ailanthus altissima*	Antioxidant Antibacterial	Romania	Yes	[73]
Cselőtey A et al., 2024	*Ailanthus altissima*	Antibacterial	Hungary	Yes	[74]
Weidner C et al., 2012	Amorfrutin	Antidiabetic	Analyticon Discovery	N/A	[75]
Kim YS et al., 2011	*Amorpha fruticosa*	Antibacterial	South Korea	Yes	[76]
Wu X et al., 2015	*Amorpha fruticosa*	Cytotoxic	China	Yes	[77]
Cui X et al., 2017	*Amorpha fruticosa*	Cytotoxic	China	Yes	[78]
Lee W et al., 2016	*Amorpha fruticosa*	Antidiabetic	South Korea	Yes	[79]
Jankovská D et al., 2024	*Amorpha fruticosa*	Anti-enzymatic	Czech Republic	Yes	[80]
Schlick J et al., 2000	GnRH-PAP	Cytotoxic	N/A	N/A	[81]
Ishag HZ et al., 2013	PAP	Antiviral	Huazhong University of Science and Technology, Wuhan, China	N/A	[82]
Mansouri S et al., 2009	PAP	Antiviral	N/A	N/A	[83]
Zhabokritsky A et al., 2014	PAP	Antiviral	N/A	N/A	[84]
Takahasi H et al., 2003	*Phytolacca americana*	Neurotrophic	Japan	Yes	[85]
Zheleva-Dimitrova Dzh et al., 2013	*Phytolacca americana*	Antioxidant Anti-enzymatic	Bulgaria	Yes	[86]
Patra JK et al., 2014	*Phytolacca americana*	Antibacterial	South Korea	Yes	[87]
Saleri FD et al., 2017	*Phytolacca americana*	Cytotoxic	China	Yes	[88]
Popovici LF et al., 2025	*Phytolacca americana*	Anxiolytic, Anti-enzymatic	Romania	Yes	[89]

N/A = not applicable; PAP = pokeweed antiviral protein; GnRH-PAP = pokeweed antiviral protein fused with gonadotropin-releasing hormone.

**Table 2:** Overview of antibacterial activity reported in selected studies, including bacterial strains tested, type of extract or isolated compound, plant material and origin, experimental model (in vitro or in vivo), and reference. Only effective results are presented, ordered after the tested bacterial strain.

Bacterial Strain	Growth Inhibition Capability	Extract Type/Isolated Compound	Plant Material/Origin	In Vivo/In Vitro	Reference
*Bacillus subtilis* ATCC 6633	IZ = 8.00	Hydroethanolic crude extract of dried autumn leaves.	*Ailanthus altissima*, leaf, Romania	In vitro	[74]
*Bacillus subtilis* F1276	MIC = 0.07	13-HODE isolated from fractionated methanolic crude young stem bark extract.	*Ailanthus altissima*, bark, Hungary	In vitro	[74]
	MIC = 0.07	9-HODE isolated from fractionated methanolic crude young stem bark extract.			
	MIC = 0.07	Juniperic acid isolated from fractionated methanolic outer trunk bark extract.			
	MIC = 0.01	Canthin-6-one isolated from fractionated methanolic inner trunk bark extract.			
*Clostridium perfringens* Neuraminidase (E.C. 3.2.1.18.)	IC_50_ = 4.15	Amoradicin isolated from the hexane–acetone fraction.	*Amorpha fruticosa*, root, South Korea	In vitro	[76]
	IC_50_ = 0.12	Amorisin isolated from the hexane–acetone subfraction.			
	IC_50_ = 7.86	Isoamoritin isolated from the hexane–acetone subfraction.			
	IC_50_ = 22.03	Amoricin from the hexane–acetone subfraction.			
	IC_50_ = 12.94	Amorphigenin from the 80% methanolic subfraction.			
	IC_50_ = 16.74	Dalbinol isolated from the hexane–acetone subfraction.			
	IC_50_ = 8.34	6-ketodehydroamorphigenin isolated from the hexane–acetone subfraction.			
*Enterococcus faecalis* ATCC 29212	IZ = 8.00	Hydroethanolic crude extract of frozen summer leaves.	*Ailanthus altissima*, leaf, Romania	In vitro	[73]
	IZ = 9.00	Hydroethanolic crude extract of frozen autumn leaves.			
	IZ = 9.00	Hydroethanolic crude extract of dried summer leaves.			
	IZ = 10.00	Hydroethanolic crude extract of dried autumn leaves.			
*Escherichia coli* ATCC 25922	MIC = 31.25	Crude 80% methanolic extract, macerated.	*Ailanthus altissima*, leaf, Blida, Algeria	In vitro	[70]
	MIC = 8.00		*Ailanthus altissima*, leaf, Tlemcen, Algeria		
	MIC = 31.25		*Ailanthus altissima*, leaf, Bizerte, Tunisia		
	MIC = 31.25		*Ailanthus altissima*, leaf, Sousse, Tunisia		
	MBC = 250.00		*Ailanthus altissima*, leaf, Blida, Algeria		
	MBC = 250.00		*Ailanthus altissima*, leaf, Tlemcen, Algeria		
	IZ = 9.00	Hydroethanolic crude extract of frozen summer leaves.	*Ailanthus altissima*, leaf, Romania	In vitro	[73]
	IZ = 10.00	Hydroethanolic crude extract of dried autumn leaves.			
*Porphyromonas gingivalis* W83 ATCC BAA-1703	MIC = 0.20	Crude 80% methanolic	*Phytolacca americana*, leaf and soft stem, South Korea	In vitro	[87]
	MIC = 0.20	Hexane fraction			
	MIC = 0.20	CHCl_3_ fraction			
*Pseudomonas aeruginosa*	BI: 29.60	Isoamoritin isolated from the hexane–acetone subfraction.	*Amorpha fruticosa*, root, South Korea	In vivo	[76]
	BI: 21.00	Dalbinol isolated from the hexane–acetone subfraction.			
*Pseudomonas* *aeruginosa* ATCC 27853	MIC = 31.25	Crude 80% methanolic extract, macerated.	*Ailanthus altissima*, leaf, Blida, Algeria	In vitro	[70]
	MIC = 16.00		*Ailanthus altissima*, leaf, Tlemcen, Algeria		
	MIC = 125.00		*Ailanthus altissima*, leaf, Bizerte, Tunisia		
	MIC = 72.50		*Ailanthus altissima*, leaf, Sousse, Tunisia		
	MBC = 250.00		*Ailanthus altissima*, leaf, Tlemcen, Algeria		
*Staphylococcus aureus* ATCC 25923	MIC = 4.00	Crude 80% methanolic	*Ailanthus altissima*, leaf, Blida, Algeria	In vitro	[70]
	MIC = 16.00		*Ailanthus altissima*, leaf, Tlemcen, Algeria		
	MIC = 4.00		*Ailanthus altissima*, leaf, Bizerte, Tunisia		
	MIC = 4.00		*Ailanthus altissima*, leaf, Sousse, Tunisia		
	MBC = 16.00		*Ailanthus altissima*, leaf, Blida, Algeria		
	MBC = 72.00		*Ailanthus altissima*, leaf, Tlemcen, Algeria		
	MBC = 16.00		*Ailanthus altissima*, leaf, Bizerte, Tunisia		
	IZ = 8.00	Hydroethanolic crude extract of dried summer leaves.	*Ailanthus altissima*, leaf, Romania	In vitro	[73]
	IZ = 8.00	Hydroethanolic crude extract of dried autumn leaves.			
*Staphylococcus aureus* Clinical isolate	IZ = 8.00	Hydroethanolic crude extract of frozen summer leaves.	*Ailanthus altissima*, leaf, Romania	In vitro	[73]
	IZ = 8.00	Hydroethanolic crude extract of frozen autumn leaves.			
	IZ = 10.00	Hydroethanolic crude extract of dried summer leaves.			
	IZ = 10.00	Hydroethanolic crude extract of dried autumn leaves.			
*Staphylococcus epidermidis* ATCC 2059	MIC = 72.25	Crude 80% methanolic extract, macerated.	*Ailanthus altissima*, leaf, Blida, Algeria	In vitro	[70]
	MIC = 8.00		*Ailanthus altissima*, leaf, Tlemcen, Algeria		
	MIC = 16.00		*Ailanthus altissima*, leaf, Bizerte, Tunisia		
	MIC = 16.00		*Ailanthus altissima*, leaf, Sousse, Tunisia		
	MBC = 125.00		*Ailanthus altissima*, leaf, Blida, Algeria		
	MBC = 125.00		*Ailanthus altissima*, leaf, Tlemcen, Algeria		
*Streptococcus mutans* UA159 ATCC 700610	MIC = 1.80 (44%)	Crude 80% methanolic	*Phytolacca americana*, leaf and soft stem, South Korea	In vitro	[87]
	MIC = 0.20	Hexane fraction			
	MIC = 0.20	CHCl_3_ fraction			
*Streptococcus pyogenes* ATCC 19615	IZ = 8.00	Hydroethanolic crude extract of dried autumn leaves.	*Ailanthus altissima*, leaf, Romania	In vitro	[73]

MIC = minimum inhibitory concentration expressed in mg/mL. MBC = minimum bactericidal concentration expressed in mg/mL. IZ = inhibition zone expressed in mm. CHCl_3_ = chloroform/trichloromethane; BI = biofilm inhibition in %; IC_50_ = half maximal inhibitory concentration expressed in μM. 13-HODE = (9Z,11E)-13-hydroxy-9,11-octadecadienoic acid. 9-HODE = (10E,12Z)-9-hydroxy-10,12-octadecadienoic acid.

**Table 3:** Summary of antiviral activity reported in selected studies, including the targeted viruses, type of extract or isolated compound, experimental model (in vitro or in vivo), and references. Only results demonstrating effective antiviral potential are included.

Virus	Result	Extract Type/Isolated Compound	Tested Cell Line/Organism	In Vivo/In Vitro	Reference
EBV	IC_50_ = 221.00	Ailantinol E from the methanolic extract of aerial part of *Ailanthus altissima*.	EBV-EA-positive Raji cells.	In vitro	[50]
	IC_50_ = 180.00	Ailantinol F from the methanolic extract of aerial part of *Ailanthus altissima*.			
	IC_50_ = 285.00	Ailantinol G from the methanolic extract of aerial part of *Ailanthus altissima*.			
HIV-1	450-fold reduction in virus production due to altering the splicing of RNAs	PAP (0.50, 1.00, or 2.50 μg)	Human embryonic kidney 293T cells transfected with a proviral clone of HIV-1.	In vitro	[84]
	HIV-1 fusion inhibition of 74.90%	*Ailanthus altissima* stem bark methanolic extract (D = 100.00 μg/mL).	HeLa-CD4+ cell lines, recombinant vaccinia virus vPE 16 with the expressed HIV-1 envelope protein gp120/41.	In vitro	[52]
HTLV-I	Suppression of HTLV-I gene expression at translational and transcriptional levels, diminishing virus production. Absence of cytotoxicity.	PAP (10.00, 25.00 and 50.00 ng)	Human embryonic kidney 293Tcells, Jur- KAT cells, and HTLV-I-infected human T-cell line.	In vitro	[83]
JEV	IC_50_ = 23.10 (inhibitory value of JEV titre)	PAP (0.10–50.00 μg/mL)	JEV-infected BHK-21 cells.	In vitro	[82]
	SP = 87.50	PAP (pre-infection, intraperitoneally, D = 1.00 mg/kg)	2-week-old BALB/c mice challenged with a lethal dose of JEV.	In vivo	
	SP = 85.70	PAP (post-infection, intraperitoneally, D = 1.00 mg/kg)			

IC_50_ = half maximal inhibitory concentration expressed in nM (unless otherwise specified). PAP = pokeweed antiviral protein, found in *Phytolacca americana*. BHK-21 cells = baby hamster kidney cells. D = dose. SP = survival percentage in %; EBV-EA = Epstein–Barr virus early antigen.

**Table 4:** Overview of antioxidant activity recorded in selected studies, including assay methods, observed results, extract types or isolated compounds, plant material and origin, experimental model (in vitro or in vivo), and reference. Only results demonstrating effective antioxidant potential are presented.

Assay Method	Result	Extract Type/Isolated Compound	Plant Material/Origin	In Vivo/In Vitro	Reference
ABTS	IC_50_ = 643.21 μg/mL	Methanolic extract	*Ailanthus altissima*, bark, Pakistan	In vitro	[69]
	c = 299.54 mmol TE/g DW	Ethanolic extract	*Ailanthus altissima*, leaf, Bulgaria	In vitro	[71]
	c = 893.14 mmol TE/g DW		*Ailanthus altissima*, flower, Bulgaria		
	c = 31.24 mmol TE/g DW		*Ailanthus altissima*, stem bark, Bulgaria		
	IC_50_ = 18.43 μg/mL	Methanolic extract	*Phytolacca americana*, leaf, Bulgaria	In vitro	[86]
	IC_50_ = 112.49 μg/mL		*Phytolacca americana*, fruit, Bulgaria		
	IC_50_ = 2.93 μg/mL	Methanolic extract	*Amorpha fruticosa*, leaf, Bulgaria	In vitro	[86]
	IC_50_ = 2.90 μg/mL		*Amorpha fruticosa*, fruit, Bulgaria		
CUPRAC	c = 548.07 mmol TE/g DW	Ethanolic extract	*Ailanthus altissima*, leaf, Bulgaria	In vitro	[71]
	c = 789.54 mmol TE/g DW		*Ailanthus altissima*, flower, Bulgaria		
	c = 10.22 mmol TE/g DW		*Ailanthus altissima*, stem bark, Bulgaria		
DPPH	IC_50_ = 88.79 μg/mL	Methanolic extract	*Phytolacca americana*, leaf, Bulgaria	In vitro	[86]
	IC_50_ = 412.06 μg/mL		*Phytolacca americana*, fruit, Bulgaria		
	IC_50_ = 16.00	Americanoic Acid A Methyl Ester	*Phytolacca americana*, seed, Japan	In vitro	[85]
	IC_50_ = 38.00	Isoamericanoic Acid A Methyl Ester			
	IC_50_ = 9.00	9′-O-Methylamericanol A			
	IC_50_ = 11.00	Americanin-type 4			
	IC_50_ = 39.00	Isoamericanin-type 5			
	IC_50_ = 5.00	Americanol A			
	IC_50_ = 16.00	Isoamericanol A			
	IC_50_ = 10.00	Americanin A			
	IC_50_ = 16.00	Isoamericanin A			
	IC_50_ = 741.74 μg/mL	Methanolic extract	*Ailanthus altissima*, bark, Pakistan	In vitro	[69]
	c = 225.62 mmol TE/g DW	Ethanolic extract	*Ailanthus altissima*, leaf, Bulgaria	In vitro	[71]
	c = 729.72 mmol TE/g DW		*Ailanthus altissima*, flower, Bulgaria		
	c = 24.96 mmol TE/g DW		*Ailanthus altissima*, stem bark, Bulgaria		
	SR = 20.95% (c = 15.63 µg/mL)	Ethanolic extract	*Ailanthus altissima*, fruit, Bozhou Baohua Pharmaceutical Co., Ltd.	In vitro	[66]
	SR = 91.97% (c = 0.50 mg/mL)				
	SR = 97.90% (c = 1.00 mg/mL)				
	IC_50_ = 11.23 μg/mL	Methanolic extract	*Amorpha fruticosa*, leaf, Bulgaria	In vitro	[86]
	IC_50_ = 9.83 μg/mL		*Amorpha fruticosa*, fruit, Bulgaria		
FIC	IP = 54.94% (c = 1 mg/mL)	Ethanolic extract	*Ailanthus altissima*, fruit, Bozhou Baohua Pharmaceutical Co., Ltd.	In vitro	[66]
FRAP	c = 906.01 mmol TE/g DW	Ethanolic extract	*Ailanthus altissima*, leaf, Bulgaria	In vitro	[71]
	c = 661.48 mmol TE/g DW		*Ailanthus altissima*, flower, Bulgaria		
	c = 16.65 mmol TE/g DW		*Ailanthus altissima*, stem bark, Bulgaria		
	IC_50_ = 508.81 μg/mL	Methanolic extract	*Amorpha fruticosa*, leaf, Bulgaria	In vitro	[86]
	IC_50_ = 642.95 μg/mL		*Amorpha fruticosa*, fruit, Bulgaria		
O_2_^−^	IC_50_ = 412.06 μg/mL	Americanoic Acid A Methyl Ester	*Phytolacca americana*, fruit, Bulgaria	In vitro	[85]
	IC_50_ = 64.00	Isoamericanoic Acid A Methyl Ester			
	IC_50_ = 8.00	9′-O-Methylamericanol A			
	IC_50_ = 9.00	Americanin-type 4			
	IC_50_ = 29.00	Isoamericanin-type 5			
	IC_50_ = 24.00	Americanol A			
	IC_50_ = 23.00	Isoamericanol A			
	IC_50_ = 9.00	Americanin A			
	IC_50_ = 58.00	Isoamericanin A			
OH	IP = 42.34% (c = 1.00 mg/mL)	Ethanolic extract	*Ailanthus altissima*, fruit, Bozhou Baohua Pharmaceutical Co., Ltd.	In vitro	[66]

IC_50_ = half maximal inhibitory concentration expressed in μM (unless specified otherwise), DPPH = 2,2-Diphenyl-1-picrylhydrazyl, O_2_^−^ = superoxide anion, c = concentration, TE = Trolox equivalents, DW = dry weight, SR = scavenging rates, IP = inhibition percentage, ABTS = 2,2′-azino-bis(3-ethylbenzothiazoline-6-sulfonic acid, CUPRAC = cupric ion-reducing antioxidant capacity, FRAP = ferric-reducing antioxidant power, FIC = ferrous ion chelating, and OH = hydroxyl.

**Table 5:** Overview of cytotoxic activity reported in selected studies, including tested cell lines or organisms, observed results, extract types or isolated compounds, assay methods, plant material and origin, experimental model (in vitro or in vivo), and reference. Only results demonstrating effective cytotoxic potential are presented, ordered after the tested cell line or organism.

Tested Cell Line/Organism	Result	Extract Type/Isolated Compound	Assay	Plant Material/Origin	In Vivo/In Vitro	Reference
2008/MRP1	IC_50_ = 7.08	Ailanthone	MTT	Puruifa Science & Technology Development Co., Chengdu, China	In vitro	[39]
A2780	IC_50_ = 10.60 μg/mL	Crude ethanolic	MTT	*Ailanthus altissima*, bark, South Korea	In vitro	[62]
	IC_50_ = 89.50 μg/mL	*n*-Hexane fraction				
	IC_50_ = 8.60 μg/mL	EtOAc fraction				
	IC_50_ = 7.10 μg/mL	*n*-BuOH fraction				
	IC_50_ = 25.70 μg/mL	Water fraction				
Cal-27	ACP = 39.00 (24 h)	Ailanthone (D = 4.00 µM)	Apoptosis	Jiangxi Herb Tiangong Technology, Jiangxi, China	In vitro	[43]
	IC_50_ = 0.84	Ailanthone	MTT			
HCT-116	IC_50_ = 1.79	Dalbin	MTT	*Amorpha fruticosa*, seed, China	In vitro	[77]
	IC_50_ = 1.98	8′-O-β-D-glucopyranosyl-amorphigenin				
	IC_50_ = 0.6	Ailanthone	CCK-8	Shanghai Yiyan Biotechnology Co., Ltd., Shanghai, China	In vitro	[42]
HEK293/R2	IC_50_ = 4.77	Ailanthone	MTT	Puruifa Science & Technology Development Co., Chengdu, China	In vitro	[39]
HeLa	CV = 6.00	Chloroform crude extract	Trypan blue staining	*Ailanthus altissima*, root, Italy	In vitro	[53]
	CV = 6.00	Chloroform fraction				
	CV = 9.00	1-Methoxy-canthin-6-one isolated from the chloroform fraction				
	ACP = 41.00 (48 h)	Chloroform crude extract	Cell apoptosis			
	ACP = 28.00 (48 h)	Chloroform fraction				
	ACP = 27.00 (48 h)	1-methoxy-canthin-6-one isolated from the chloroform fraction				
Hep3B	IC_50_ = 0.37	Altissinol A	MTT	*Ailanthus altissima*, bark, China	In vitro	[56]
	IC_50_ = 0.48	Ailanthone				
	IC_50_ = 0.98	13,18-Dehydroglaucarubinone				
	IC_50_ = 2.17	(-)-Cha-parrinone				
	IC_50_ = 0.05	6*a*-Tigloyloxychaparrinone				
	IC_50_ = 8.01	Shinjulactone A				
	IC_50_ = 2.36	Altissinol B				
	IC_50_ = 2.43	6*a*-Tigloyloxychaparrin				
	IC_50_ = 23.46	Glaucarubin				
	IC_50_ = 47.08	Chouchunsteride B	MTT	*Ailanthus altissima*, leaf, China	In vitro	[68]
	IC_50_ = 31.52	Chouchunsteride D				
	IC_50_ = 31.49	6-dehydropregnenolone				
	IC_50_ = 44.52	20*S*- Hydroxyergosta-4,6,24(28)-trien-3-one				
	IC_50_ = 39.88	3-O-β-*D*-glucopyranosyl-16-dehydropregnenolone				
	IC_50_ = 45.21	Altissimacoumarin C	MTT	*Ailanthus altissima*, root bark, China	In vitro	[65]
	IC_50_ = 0.54	Ailanthone	MTT	Jinan University, Guangzhou, China	In vitro	[37]
HepG2	IC_50_ = 0.28	Altissinol A	MTT	*Ailanthus altissima*, bark, China	In vitro	[56]
	IC_50_ = 0.24	Ailanthone				
	IC_50_ = 1.15	13,18-Dehydroglaucarubinone				
	IC_50_ = 1.20	(-)-Cha-parrinone				
	IC_50_ = 0.55	6a-Tigloyloxychaparrinone				
	IC_50_ = 4.67	Shinjulactone A				
	IC_50_ = 1.22	Altissinol B				
	IC_50_ = 10.54	6a-Tigloyloxychaparrin				
	IC_50_ = 35.57	Glaucarubin				
	IC_50_ = 4.03	Chouchunsteride A	MTT	*Ailanthus altissima*, leaf, China	In vitro	[68]
	IC_50_ = 7.62	6-dehydropregnenolone				
	IC_50_ = 13.43	20*S*- Hydroxyergosta-4,6,24(28)-trien-3-one				
	IC_50_ = 66.47	(+)-7S,8R-ailanthussin A	MTT	*Ailanthus altissima*, bark, China	In vitro	[61]
	IC_50_ = 29.53	(−)-7R,8S-ailanthussin A				
	IC_50_ = 0.63	Ailanthone	MTT	Jinan University, Guangzhou, China	In vitro	[37]
HepG2/ADM	IC_50_ = 4.03	Chouchunsteride A	MTT	*Ailanthus altissima*, leaf, China	In vitro	[56]
	IC_50_ = 7.62	6-Dehydropregnenolone				
	IC_50_ = 13.43	20*S*- Hydroxyergosta-4,6,24(28)-trien-3-one				
HL-60	IC_50_ = 5.99	Ailanthone	MTT	Jinan University, Guangzhou, China	In vitro	[38]
	ACP = 42.02	Ailanthone (5.00 μM)	Cell apoptosis			
	ACP = 52.05	Ailanthone (10.00 μM)				
	ACP = 56.69	Ailanthone (20.00 μM)				
Huh7	IC_50_ = 0.35	Ailanthone	MTT	Jinan University, Guangzhou, China	In vitro	[37]
Ishikawa cell line	IC_50_ = 3.00 nM	GnRH-PAP	Translation	N/A	In vitro	[81]
K562/A02	IC_50_ = 2.21	Ailanthone	MTT	Puruifa Science & Technology Development Co., Chengdu, China	In vitro	[39]
MCF-7	IC_50_ = 3.90	Amorphasidase	MTT	*Amorpha fruticosa*, seed, China	In vitro	[86]
	IC_50_ = 1.50	Dalbin				
	IC_50_ = 0.45	8′-O-β-D-glucopyranosyl-amorphigenin				
	IC_50_ = 0.95	Amorphin				
	IC_50_ = 34.08	6′-O-β-D-glucopyranosyl-12a-hydroxydalpanol				
	CV = 64.36	Ailanthone (0.50 µg/mL)	MTT	*Ailanthus altissima*, bark, China	In vitro	[60]
	CV = 62.48	Ailanthone (1.00 µg/mL)				
	CV = 57.64	Ailanthone (2.00 µg/mL)				
	CV = 50.24	Ailanthone (4.00 µg/mL)				
	CV = 43.24	Ailanthone (8.00 µg/mL)				
	ACP = 22.68	Ailanthone (0.50 µg/mL)	Cell apoptosis			
	ACP = 27.99	Ailanthone (1.00 µg/mL)				
	ACP = 35.88	Ailanthone (2.00 µg/mL)				
	ACP = 49.77	Ailanthone (4.00 µg/mL)				
	ACP = 75.51	Ailanthone (8.00 µg/mL)				
	BBR = 0.53	Ailanthone (0.50 µg/mL)	Bax/Bcl-2 protein expression levels			
	BBR = 0.56	Ailanthone (1.00 µg/mL)				
	BBR = 0.80	Ailanthone (2.00 µg/mL)				
	BBR = 0.93	Ailanthone (4.00 µg/mL)				
	BBR = 1.25	Ailanthone (8.00 µg/mL)				
NCM460	IC_50_ = 1.76	Ailanthone	CCK-8	Shanghai Yiyan Biotechnology Co., Ltd., Shanghai, China	In vitro	[42]
NSCLC Lewis cells	IC_50_ = 7.70 (24 h)	Ailanthone	MTT	Chengdu Alfa Biological Technology Co., Ltd., Chengdu, China	In vitro	[47]
OVCAR3	IC_50_ = 34.70 μg/mL	Crude ethanolic	MTT	*Ailanthus altissima*, bark, South Korea	In vitro	[62]
	IC_50_ = 28.00 μg/mL	EtOAc fraction				
	IC_50_ = 22.50 μg/mL	*n*-BuOH fraction				
	IC_50_ = 44.20 μg/mL	Water fraction				
SKOV3	IC_50_ = 34.70 μg/mL	Crude ethanolic	MTT	*Ailanthus altissima*, bark, South Korea	In vitro	[62]
	IC_50_ = 28.00 μg/mL	EtOAc fraction				
	IC_50_ = 22.50 μg/mL	*n*-BuOH fraction				
	IC_50_ = 44.20 μg/mL	Water fraction				
SW620	IC_50_ = 1.01	Ailanthone	CCK-8	Shanghai Yiyan Biotechnology Co., Ltd., Shanghai, China	In vitro	[42]
Tca8113	ACP = 17.00 (24 h)	Ailanthone (D = 4.00 µM)	Apoptosis	Jiangxi Herb Tiangong Technology, Jiangxi, China	In vitro	[43]
	IC_50_ = 0.79	Ailanthone	MTT			
U87MG	ACP = 20.00 (48 h)	Chloroform crude extract	Cell apoptosis	*Ailanthus altissima*, root, Italy	In vitro	[53]
	ACP = 8.00 (48 h)	Chloroform fraction				
	ACP = 9.00 (48 h)	1-methoxy-canthin-6-one isolated from the chloroform fraction				
U937	ACP = 19.00 (48 h)	Chloroform crude extract	Cell apoptosis			
	ACP = 11.00 (48 h)	Chloroform fraction				
	ACP = 11.00 (48 h)	1-methoxy-canthin-6-one isolated from the chloroform fraction				

GnRH-PAP = gonadotropin-releasing hormone fused with pokeweed antiviral protein, MTT = 3-[4,5-dimethylthiazol-2-yl]-2,5 diphenyl tetrazolium bromide, MCF-7 = breast cancer cell line, IC_50_ = half maximal inhibitory concentration expressed in μM (unless specified otherwise), HCT-116 = human colorectal carcinoma cell line, CCK-8 = cell counting kit-8, Hep3B = human hepatoma cell line, HepG2 = human hepatoma cell line, HepG2/ADM = multidrug resistant human hepatoma cell line, CV = cell viability expressed in percentage after 72 h of treatment (unless specified otherwise), ACP = apoptotic cells percentage after 48 h of treatment (unless specified otherwise), BBR = Bax/Bcl2 ratio, HCT-116 = colon cancer cell line, A2780, SKOV3, OVCAR3 = ovarian cancer cell lines, HeLa = immortalized regular human cell line, U87MG = glioblastoma cell line, U937 = histiocytic lymphoma cell line, Tca8113, Cal-27 = squamous cell carcinoma of the human tongue cell lines, SW620 = colon cancer cell line, NCM460 = normal human colon mucosal epithelial cells, NSCLC Lewis cells = non-small cell lung cancer cell line, HL-60 = human promyelocytic leukaemia cell line, Huh7 = immortalized human hepatoma cell line, K562/A02 = doxorubicin-resistant human leukaemia cell line, 2008/MRP1 = MRP1 (multidrug resistance-associated protein 1)-overexpressing derivative of human ovarian carcinoma cell line, HEK293/R2 = human embryonic kidney 293 cell lines transfected with BCRP (breast cancer resistance protein), EtOAc = ethyl acetate, n-BuOH = n-butanol, and N/A = not applicable.

**Table 6:** Summary of enzyme inhibition, anti-inflammatory, and neuroprotective activities identified in selected studies, detailing assay types, biological effects, tested extracts or isolated compounds, plant material and origin, experimental model (in vitro or in vivo), and reference. Only results demonstrating significant biological activity are included.

Activity	Assay	Result	Extract Type/Isolated Compound	Plant Material/Origin	In Vivo/In Vitro	Reference
Neuroprotective	MTT assay on H_2_O_2_-induced SH-SY5Y cells	CV = 70.50%	7*S*,8*R*-Guaiacylglycerol-8-acetovanillone ether (50.00 μM)	*Ailanthus altissima* root bark*,* China	In vitro	[64]
Enzyme inhibition	AChE inhibition	IP = 76.00	Methanolic crude extract	*Amorpha fruticosa*, flower, Czech Republic	In vitro	[80]
		IP = 9.10	(E)-N6-(*Z*)-di-*p*-Coumaroylputrescine (100.00 μM)			
		IP = 1.10	N1,N6-(*E*)-di-*p*-Coumaroylputrescine (100.00 μM)			
		IP = 14.10	N1-(*E*)-N5,N10-(*Z*)-tri-*p*-Coumaroylspermidine (100.00 μM)			
		IP = 26.60	N1,N5-(*Z*)-N10-(E)-tri-*p*-Coumaroylspermidine (100.00 μM)			
		IP = 47.90	N1,N5,N10-(E)-tri-*p*-Coumaroylspermidine (100.00 μM)			
		IP = 17.90	*cis*-12*a*-Hydroxymunduserone (100.00 μM)			
		IP = 37.90	6-Deoxyclitoriacetal (100.00 μM)			
		IP = 22.40	12α-Hydroxy-α-toxicarol (100.00 μM)			
		IP = 25.43	Methanolic crude extract (0.17 mg/mL)	*Amorpha fruticosa*, leaf, Bulgaria	In vitro	[86]
		IP = 48.86		*Amorpha fruticosa*, fruit, Bulgaria		
		IC_50_ = 3.28	Chouchunionone A	*Ailanthus altissima*, leaf, China	In vitro	[72]
		IC_50_ = 16.31 μg/mL	Methanolic crude extract	*Ailanthus altissima*, bark, Pakistan	In vitro	[69]
	BuChE inhibition	IP = 90.00	Methanolic crude extract	*Amorpha fruticosa*, flower, Czech Republic	In vitro	[80]
		IP = 8.10	(*E*)-N6-(*Z*)-di-*p*-Coumaroylputrescine (100.00 μM)			
		IP = 4.80	N1,N6-(*E*)-di-*p*-Coumaroylputrescine (100.00 μM)			
		IP = 4.50	N1-(*E*)-N5,N10-(*Z*)-tri-*p*-Coumaroylspermidine (100.00 μM)			
		IP = 19.10	N1,N5-(*Z*)-N10-(*E*)-tri-p-Coumaroylspermidine (100.00 μM)			
		IP = 43.80	N1,N5,N10-(*E*)-tri-*p*-Coumaroylspermidine (100.00 μM)			
		IP = 23.80	*cis*-12*a*-Hydroxymunduserone (100.00 μM)			
		IP = 25.60	6-Deoxyclitoriacetal (100.00 μM)			
		IP = 37.60	Amorphispironone (100.00 μM)			
		IP = 23.60	Tephrosin (100.00 μM)			
		IP = 22.80	12α-Hydroxy-α-toxicarol (100.00 μM)			
	Tyrosinase inhibition	IC_50_ = 20.28	Chouchunionone C	*Ailanthus altissima*, leaf, China	In vitro	[72]
Anti-inflammatory	NO production inhibition in LPS-induced RAW 264.7 cells	IC_50_ = 15.09	(*R*)-5-(1-Hydroxyethyl)-canthin-6-on	*Ailanthus altissima*, stem bark, South Korea	In vitro	[58]
		IC_50_ = 9.09	Canthin-6-one			
		IC_50_ = 7.73	9-Hydroxycanthin-6-one			
		IC_50_ = 12.01	10-Hydroxycanthin-6-one			
		IC_50_ = 5.92	Sinapaldehyde			
		IC_50_ = 10.69	Erythro-guaiacylglycerol-β-O-41-coniferyl ether			
		IC_50_ = 63.50	Canthin-6-one-1-O-*b*-*D*-apiofuranosyl-(1->2)-*b*-*D*-glucopyranoside	*Ailanthus altissima*, stem bark, South Korea	In vitro	[57]
		IC_50_ = 85.00	Canthin-6-one-1-O-[6-O-(3-hydroxy-3-methylglutaryl)]-*b*- D-glucopyranoside			
		IC_50_ = 63.10	Shinjudilactone			
		IC_50_ = 5.18	Ailanthone			
		IC_50_ = 56.40	Shinjulactone A			
		IC_50_ = 72.80	4-Hydroxybenzoic acid			
		IC_50_ = 23.20	Vanillic acid			
		IC_50_ = 43.80	3-Hydroxy-1-(4-hydroxy-3-methoxyphenyl)-propan-1-one			
		IC_50_ = 73.40	*p*-coumaric acid			
		IC_50_ = 71.60	trans-4-O-*b*-*D*-Glucopyranosyl ferulic acid			
		IC_50_ = 21.60	Syringaresinol			
	COX-2 inhibition in BMMC cells	IC_50_ = 47.40 µg/mL	Ethanol crude extract	*Ailanthus altissima*, leaf and branch, South Korea	In vitro	[54]
	COX-1 inhibition in BMMC cells	IC_50_ = 131.66 µg/mL				
	LTC4 suppression in BMMC cells	IC_50_ = 25.70 µg/mL				
	β-HEX release inhibition in BMMC cells	IC_50_ = 27.30 µg/mL				

AChE = acetylcholinesterase, IP = inhibition percent expressed in %, BuChE = butyrylcholinesterase, IC_50_ = half maximal inhibitory concentration expressed in μM (unless specified otherwise), NO = nitric oxide, LPS = lipopolysaccharide, RAW 264.7 = murine macrophage cell line, COX-2 = cyclooxygenase-2, COX-1 = cyclooxygenase-1, LTC4 = leukotriene C4, BMMC = primary bone marrow mononuclear spherical cells, β-HEX = β-hexosaminidase, SH-SY5Y = human neuroblastoma cell line, and CV = cell viability.

## Data Availability

No new data was created for this manuscript.

## Abbreviations

The following abbreviations are used in this manuscript:

13-HODE	(9Z,11E)-13-hydroxy-9,11-octadecadienoic acid
9-HODE	(10E,12Z)-9-hydroxy-10,12-octadecadienoic acid
ABTS	2,2′-azino-bis(3-ethylbenzothiazoline-6-sulfonic acid
AChE	Acetylcholinesterase
ACP	Apoptotic cells percentage
BBR	Bax/Bcl2 ratio
BI	Biofilm inhibition
BMMC	Primary bone marrow mononuclear spherical cells
BuChE	Butyrylcholinesterase
C	Concentration
CAT	Catalase
CCK-8	Cell counting kit-8
CDDP	Cisplatin
CDK4	Cyclin-dependent kinase 4
COX	Cyclooxygenase
CUPRAC	Cupric ion-reducing antioxidant capacity
CV	Cell viability
D	Dose
DGF	5,7-dihydroxy-6-geranylflavanone
DMSO	Dimethyl sulfoxide
DNA	Deoxyribonucleic acid
DPPH	2,2-Diphenyl-1-picrylhydrazyl
DW	Dry weight
EBV	Epstein–Barr virus
EBV-EA	Epstein–Barr virus early antigen
ERK	Extracellular signal-regulated kinase
EU	European Union
FIC	Ferrous ion chelating
FRAP	Ferric-reducing antioxidant power
GAS5	Growth arrest specific 5
GnRH-PAP	Pokeweed antiviral protein fused with gonadotropin-releasing hormone
GSH-Px	Glutathione peroxidase
HIV-1	Human immunodeficiency virus 1
HTLV-I	Human T-cell leukaemia virus I
IL	Interleukin
iNOS	Inducible nitric oxide synthase
INPS	Invasive non-native plant species
IP	Inhibition percentage
IPNI	International Plant Names Index
IR-Akt	Insulin receptor-protein kinase B
IZ	Inhibition zone
JAK	Janus kinase
JEV	Japanese encephalitis virus
JNK	c-Jun NH2-terminal kinase
LPS	Lipopolysaccharide
LTC4	Leukotriene C4
MAPK	Mitogen-activated protein kinase
MBC	Minimum bactericidal concentration
MDA	Malondialdehyde
MIC	Minimum inhibitory concentration
mRNA	Messenger ribonucleic acid
MTT	3-[4,5-dimethylthiazol-2-yl]-2,5 diphenyl tetrazolium bromide
N/A	Not applicable
NF-κB	Nuclear factor kappa B
NO	Nitric oxide
NSLCL	Non-small cell lung cancer
OH	Hydroxyl
PAP	Pokeweed antiviral protein
PPAR	Peroxisome proliferator-activated receptor
PRISMA	Preferred Reporting Items for Systematic Reviews and Meta-Analyses
RANKL	Receptor activator of nuclear factor kappa B ligand
ROS	Reactive oxygen species
SOD	Superoxide dismutase
SP	Survival percentage
SR	Scavenging rates
STAT3	Signal transducer and activator of transcription 3
TE	Trolox equivalents
TEAD	Transcription factor
TNF	Tumour necrosis factor
TUNEL	Terminal deoxynucleotidyl transferase (TdT) dUTP nick-end labelling
UCHL1	Ubiquitin carboxy-terminal hydrolase L1
ULK1	Unc51-like autophagy activating kinase 1
UPF1	Up-frameshift protein 1
UV	Ultraviolet
WFO	World Flora Online
YAP	Yes-associated protein 1
β-HEX	β-hexosaminidase

## References

[1] Royal Horticultural Society Invasive Non-Native Plants: Prevention and Protection. Royal Horticultural Society. https://www.rhs.org.uk/prevention-protection/invasive-non-native-plants.

[2] Pyšek P., Jarošík V., Hulme P.E., Pergl J., Hejda M., Schaffner U., Vilà M. (2012). A global assessment of invasive plant impacts on resident species, communities and ecosystems: The interaction of impact measures, invading species’ traits and environment. Glob. Change Biol..

[3] Anderson L.G., Rocliffe S., Haddaway N.R., Dunn A.M. (2015). The Role of Tourism and Recreation in the Spread of Non-Native Species: A Systematic Review and Meta-Analysis. PLoS ONE.

[4] Van Kleunen M., Dawson W., Essl F., Pergl J., Winter M., Weber E., Kreft H., Weigelt P., Kartesz J., Nishino M. (2015). Global exchange and accumulation of non-native plants. Nature.

[5] Gioria M., Hulme P.E., Richardson D.M., Pyšek P. (2023). Why Are Invasive Plants Successful?. Annu. Rev. Plant Biol..

[6] Weidlich E.W.A., Flórido F.G., Sorrini T.B., Brancalion P.H.S. (2020). Controlling invasive plant species in ecological restoration: A global review. J. Appl. Ecol..

[7] Pyšek P., Hulme P.E., Simberloff D., Bacher S., Blackburn T.M., Carlton J.T., Dawson W., Essl F., Foxcroft L.C., Genovesi P. (2020). Scientists’ warning on invasive alien species. Biol. Rev..

[8] Li Y., Liu X., Zeng H., Zhang J., Zhang L. (2021). Public education improves farmers knowledge and management of invasive alien species. Biol. Invasions.

[9] Lorenzo P., Morais M.C. (2023). Strategies for the Management of Aggressive Invasive Plant Species. Plants.

[10] Invasive Alien Species—European Commission. https://environment.ec.europa.eu/topics/nature-and-biodiversity/invasive-alien-species_en.

[11] Dehnen-Schmutz K., Novoa A., Clements D.R., Upadhyaya M.K., Joshi S., Shrestha A. (2022). Advances in the Management of Invasive Plants. Global Plant Invasions.

[12] Cuthbert R.N., Diagne C., Hudgins E.J., Turbelin A., Ahmed D.A., Albert C., Bodey T.W., Briski E., Essl F., Haubrock P.J. (2022). Biological invasion costs reveal insufficient proactive management worldwide. Sci. Total Environ..

[13] Haubrock P.J., Turbelin A.J., Cuthbert R.N., Novoa A., Taylor N.G., Angulo E., Ballesteros-Mejia L., Bodey T.W., Capinha C., Diagne C. (2021). Economic costs of invasive alien species across Europe. NeoBiota.

[14] Máximo P., Ferreira L.M., Branco P.S., Lourenço A. (2020). Invasive Plants: Turning Enemies into Value. Molecules.

[15] Peter A., Žlabur J.Š., Šurić J., Voća S., Purgar D.D., Pezo L., Voća N. (2021). Invasive Plant Species Biomass—Evaluation of Functional Value. Molecules.

[16] Ahmed A., Abu Bakar M.S., Hamdani R., Park Y.-K., Lam S.S., Sukri R.S., Hussain M., Majeed K., Phusunti N., Jamil F. (2020). Valorization of underutilized waste biomass from invasive species to produce biochar for energy and other value-added applications. Environ. Res..

[17] Nunes L.J.R., Rodrigues A.M., Loureiro L.M.E.F., Sá L.C.R., Matias J.C.O. (2021). Energy Recovery from Invasive Species: Creation of Value Chains to Promote Control and Eradication. Recycling.

[18] Míguez C., Cancela Á., Álvarez X., Sánchez Á. (2022). The reuse of bio-waste from the invasive species Tradescantia fluminensis as a source of phenolic compounds. J. Clean. Prod..

[19] Banunle A., Fei-Baffoe B., Miezah K., Ewusi-Mensah N., Jørgensen U., Aidoo R., Amoah A., Addo-Fordjour P., Abaidoo R.C. (2023). Valorisation of Biowaste and Aquatic Invasive Plants Through Compost Production for Agricultural Use. Waste Biomass Valorization.

[20] Lorenzo P., Morais M.C. (2024). Repurposing Waste from Aggressive Acacia Invaders to Promote Its Management in Large Invaded Areas in Southwestern Europe. Plants.

[21] Raudone L., Savickiene N. (2024). Phytochemical Profiles of Plant Materials: From Extracts to Added-Value Ingredients. Plants.

[22] Vrabič-Brodnjak U., Možina K. (2022). Invasive Alien Plant Species for Use in Paper and Packaging Materials. Fibers.

[23] Quinty V., Colas C., Nasreddine R., Nehmé R., Piot C., Draye M., Destandau E., Da Silva D., Chatel G. (2022). Screening and Evaluation of Dermo-Cosmetic Activities of the Invasive Plant Species Polygonum cuspidatum. Plants.

[24] Chopra B., Dhingra A.K. (2021). Natural products: A lead for drug discovery and development. Phytother. Res..

[25] Dzobo K. (2022). The Role of Natural Products as Sources of Therapeutic Agents for Innovative Drug Discovery. Comprehensive Pharmacology.

[26] Luo Z., Yin F., Wang X., Kong L. (2024). Progress in approved drugs from natural product resources. Chin. J. Nat. Med..

[27] Cappuccino N., Arnason J.T. (2006). Novel chemistry of invasive exotic plants. Biol. Lett..

[28] Macel M., De Vos R.C.H., Jansen J.J., Van Der Putten W.H., Van Dam N.M. (2014). Novel chemistry of invasive plants: Exotic species have more unique metabolomic profiles than native congeners. Ecol. Evol..

[29] Skubel S.A., Su X., Poulev A., Foxcroft L.C., Dushenkov V., Raskin I. (2020). Metabolomic differences between invasive alien plants from native and invaded habitats. Sci. Rep..

[30] Sirbu C., Miu I.V., Gavrilidis A.A., Gradinaru S.R., Niculae I.M., Preda C., Oprea A., Urziceanu M., Camen-Comanescu P., Nagoda E. (2022). Distribution and pathways of introduction of invasive alien plant species in Romania. NeoBiota.

[31] Anastasiu P., Miu I.V., Gavrilidis A.A., Preda C., Rozylowicz L., Sirbu C., Oprea A., Urziceanu M., Camen-Comanescu P., Nagoda E. (2024). Alien plant species distribution in Romania: A nationwide survey following the implementation of the EU Regulation on Invasive Alien Species. Biodivers. Data J..

[32] Dumitrascu M., Grigorescu I., Doroftei M., Kucsicsa G., Mierla M., Dragota C.-S., Nastase M. (2013). Assessing Invasive Terrestrial Plan Species *Amorpha fruticosa* in Three Wetland Areas in Romania: Danube Delta Biosphere Reserve, Comana Natural Park and Mures Floodplain Natural Park. Int. Multidiscip. Sci. GeoConf. SGEM.

[33] Plants of the World Online|Kew Science. https://powo.science.kew.org/.

[34] International Plant Names Index. https://www.ipni.org/.

[35] The WFO Plant List|World Flora Online. https://wfoplantlist.org/.

[36] Ouzzani M., Hammady H., Fedorowicz Z., Elmagarmid A. (2016). Rayyan—A web and mobile app for systematic reviews. Syst. Rev..

[37] Zhuo Z., Hu J., Yang X., Chen M., Lei X., Deng L., Yao N., Peng Q., Chen Z., Ye W. (2015). Ailanthone Inhibits Huh7 Cancer Cell Growth via Cell Cycle Arrest and Apoptosis In Vitro and In Vivo. Sci. Rep..

[38] Wei C., Chen C., Cheng Y., Zhu L., Wang Y., Luo C., He Y., Yang Z., Ji Z. (2018). Ailanthone induces autophagic and apoptotic cell death in human promyelocytic leukemia HL-60 cells. Oncol. Lett..

[39] Han F., Liu G., Sun C., Wei J. (2018). Ailanthone reverses multidrug resistance by inhibiting the P-glycoprotein-mediated efflux in resistant K562/A02 cells. Cell. Mol. Biol..

[40] Daga M., Pizzimenti S., Dianzani C., Cucci M.A., Cavalli R., Grattarola M., Ferrara B., Scariot V., Trotta F., Barrera G. (2019). Ailanthone inhibits cell growth and migration of cisplatin resistant bladder cancer cells through down-regulation of Nrf2, YAP, and c-Myc expression. Phytomedicine.

[41] Cucci M.A., Grattarola M., Dianzani C., Damia G., Ricci F., Roetto A., Trotta F., Barrera G., Pizzimenti S. (2020). Ailanthone increases oxidative stress in CDDP-resistant ovarian and bladder cancer cells by inhibiting of Nrf2 and YAP expression through a post-translational mechanism. Free Radic. Biol. Med..

[42] Ding H., Yu X., Yan Z. (2021). Ailanthone suppresses the activity of human colorectal cancer cells through the STAT3 signaling pathway. Int. J. Mol. Med..

[43] Wang S., Cui Q., Chen X., Zhu X., Lin K., Zheng Q., Wang Y., Li D. (2022). Ailanthone Inhibits Cell Proliferation in Tongue Squamous Cell Carcinoma via PI3K/AKT Pathway. Evid. Based Complement. Alternat. Med..

[44] Zhang Y., Gong R., Liu Y., Sun X., Liang J., Zhou Y., Wang Y., Yu W., Wang Y., Tang L. (2022). Ailanthone Inhibits Proliferation, Migration and Invasion of Osteosarcoma Cells by Downregulating the Serine Biosynthetic Pathway. Front. Oncol..

[45] Wang Y., Zhong Z., Ma M., Zhao Y., Zhang C., Qian Z., Wang B. (2023). The role played by ailanthone in inhibiting bone metastasis of breast cancer by regulating tumor-bone microenvironment through the RANKL-dependent pathway. Front. Pharmacol..

[46] Fang C., Wu W., Ni Z., Liu Y., Luo J., Zhou Y., Gong C., Hu D., Yao C., Chen X. (2024). Ailanthone inhibits non-small cell lung cancer growth and metastasis through targeting UPF1/GAS5/ULK1 signaling pathway. Phytomedicine.

[47] Yang H., Zhang X., Lu Y., Wang X., Zhang Z., Xu H., Li F., Chen Q., Bai Y., Bai X. (2024). Ailanthone induces autophagy and ferroptosis in non-small cell lung cancer Lewis cells. Mol. Clin. Oncol..

[48] Liang J., Qiao G., Zhang Y., Yuan Y., Liu Z., Jiang Y., Zhang Y., Deng Z., Yu L., Lin H. (2025). Ailanthone targets the KMT2A-MEN1 complex to suppress lung metastasis of osteosarcoma. Phytomedicine.

[49] Li J., Lv Y., Xue S., Li W., Zhang X. (2025). Ailanthone inhibits bladder cancer tumor and cell proliferation, epithelial-mesenchymal transition, and activation of the Janus kinase/signal transducer and activator of transcription 3 signaling pathway. Cytojournal.

[50] Tamura S., Fukamiya N., Okano M., Koyama J., Koike K., Tokuda H., Aoi W., Takayasu J., Kuchide M., Nishino H. (2003). Three New Quassinoids, Ailantinol E, F, and G, from *Ailanthus altissima*. Chem. Pharm. Bull..

[51] Okunade A.L., Bikoff R.E., Casper S.J., Oksman A., Goldberg D.E., Lewis W.H. (2003). Antiplasmodial activity of extracts and quassinoids isolated from seedlings of *Ailanthus altissima* (Simaroubaceae). Phytother. Res..

[52] Chang Y., Woo E. (2003). Korean medicinal plants inhibiting to Human Immunodeficiency Virus type 1 (HIV-1) fusion. Phytother. Res..

[53] De Feo V., Martino L.D., Santoro A., Leone A., Pizza C., Franceschelli S., Pascale M. (2005). Antiproliferative effects of tree-of-heaven (*Ailanthus altissima* Swingle). Phytother. Res..

[54] Jin M.H., Yook J., Lee E., Lin C.X., Quan Z., Son K.H., Bae K.H., Kim H.P., Kang S.S., Chang H.W. (2006). Anti-inflammatory Activity of *Ailanthus altissima* in Ovalbumin-Induced Lung Inflammation. Biol. Pharm. Bull..

[55] Ammirante M., Di Giacomo R., De Martino L., Rosati A., Festa M., Gentilella A., Pascale M.C., Belisario M.A., Leone A., Caterina Turco M. (2006). 1-Methoxy-Canthin-6-One Induces c-Jun NH2-Terminal Kinase–Dependent Apoptosis and Synergizes with Tumor Necrosis Factor–Related Apoptosis-Inducing Ligand Activity in Human Neoplastic Cells of Hematopoietic or Endodermal Origin. Cancer Res..

[56] Wang Y., Wang W.-J., Su C., Zhang D.-M., Xu L.-P., He R.-R., Wang L., Zhang J., Zhang X.-Q., Ye W.-C. (2013). Cytotoxic quassinoids from *Ailanthus altissima*. Bioorg. Med. Chem. Lett..

[57] Kim H.M., Kim S.J., Kim H.-Y., Ryu B., Kwak H., Hur J., Choi J.-H., Jang D.S. (2015). Constituents of the stem barks of *Ailanthus altissima* and their potential to inhibit LPS-induced nitric oxide production. Bioorg. Med. Chem. Lett..

[58] Kim H., Lee J., Sezirahiga J., Kwon J., Jeong M., Lee D., Choi J.-H., Jang D. (2016). A New Canthinone-Type Alkaloid Isolated from *Ailanthus altissima* Swingle. Molecules.

[59] He Q., Xiao H., Li J., Liu Y., Jia M., Wang F., Zhang Y., Wang W., Wang S. (2018). Fingerprint analysis and pharmacological evaluation of *Ailanthus altissima*. Int. J. Mol. Med..

[60] Wang R., Lu Y., Li H., Sun L., Yang N., Zhao M., Zhang M., Shi Q. (2018). Antitumor activity of the *Ailanthus altissima* bark phytochemical ailanthone against breast cancer MCF-7 cells. Oncol. Lett..

[61] Yan Z.-Y., Chen J.-J., Duan Z.-K., Yao G.-D., Lin B., Wang X.-B., Huang X.-X., Song S.-J. (2018). Racemic phenylpropanoids from the root barks of *Ailanthus altissima* (Mill.) Swingle with cytotoxicity against hepatoma cells. Fitoterapia.

[62] Jeong M., Kim H.M., Ahn J.-H., Lee K.-T., Jang D.S., Choi J.-H. (2018). 9-Hydroxycanthin-6-one isolated from stem bark of *Ailanthus altissima* induces ovarian cancer cell apoptosis and inhibits the activation of tumor-associated macrophages. Chem. Biol. Interact..

[63] Rahman H.M.A., Rasool M.F., Imran I. (2019). Pharmacological Studies Pertaining to Smooth Muscle Relaxant, Platelet Aggregation Inhibitory and Hypotensive Effects of *Ailanthus altissima*. Evid. Based Complement. Alternat. Med..

[64] Du Y.-Q., Lin B., Yan Z.-Y., Hou Z.-L., Guo R., Bai M., Zhou L., Huang X.-X., Song S.-J. (2019). Enantiomeric 8,4′-type oxyneolignans from the root barks of *Ailanthus altissima* (Mill.) Swingle and their neuroprotective effects against H_2_O_2_-induced SH-SY5Y cells injury. Fitoterapia.

[65] Yan Z.-Y., Lv T.-M., Wang Y.-X., Shi S.-C., Chen J.-J., Bin-Lin, Liu Q.-B., Huang X.-X., Song S.-J. (2020). Terpenylated coumarins from the root bark of *Ailanthus altissima* (Mill.) Swingle. Phytochemistry.

[66] Mo Y., Cheng F., Yang Z., Shang X., Liang J., Shang R., Hao B., Wang X., Zhang H., Wali A. (2021). Antioxidant Activity and the Potential Mechanism of the Fruit from *Ailanthus altissima* Swingle. Front. Vet. Sci..

[67] Kim S.R., Park Y., Li M., Kim Y.K., Lee S., Son S.Y., Lee S., Lee J.S., Lee C.H., Park H.H. (2022). Anti-inflammatory effect of *Ailanthus altissima* (Mill.) Swingle leaves in lipopolysaccharide-stimulated astrocytes. J. Ethnopharmacol..

[68] Gao Z.-H., Duan Z.-K., Ma Z.-T., Ye L., Yao G.-D., Huang X.-X., Song S.-J. (2022). Chouchunsteride A–D, four new steroids from the leaves of *Ailanthus altissima* (Mill.) Swingle. Steroids.

[69] Muhammad Abdur Rahman H., Javaid S., Ashraf W., Fawad Rasool M., Saleem H., Ali Khan S., Ul-Haq Z., Muhammad Muneeb Anjum S., Ahmad T., Alqahtani F. (2023). Effects of long-term *Ailanthus altissima* extract supplementation on fear, cognition and brain antioxidant levels. Saudi Pharm. J..

[70] Boukhibar H., Laouani A., Touzout S.N., Alenazy R., Alqasmi M., Bokhari Y., Saguem K., Ben-Attia M., El-Bok S., Merghni A. (2023). Chemical Composition of *Ailanthus altissima* (Mill.) Swingle Methanolic Leaf Extracts and Assessment of Their Antibacterial Activity through Oxidative Stress Induction. Antibiotics.

[71] Andonova T., Muhovski Y., Slavov I., Vrancheva R., Georgiev V., Apostolova E., Naimov S., Mladenov R., Pavlov A., Dimitrova-Dyulgerova I. (2023). Phenolic Profile, Antioxidant and DNA-Protective Capacity, and Microscopic Characters of *Ailanthus altissima* Aerial Substances. Plants.

[72] Song Q., Duan Z.-K., Tan Y.-N., Gao Z.-H., Liu D., Hao J.-L., Lin B., Huang X.-X., Song S.-J. (2024). Isolation of four new monoterpenes from *Ailanthus altissima* (Mill.) Swingle and their enzyme inhibitory effects. Fitoterapia.

[73] Cocîrlea M.D., Soare A., Petrovici A.R., Silion M., Călin T., Oancea S. (2024). Phenolic Composition and Bioactivities of Invasive *Ailanthus altissima* (Mill.) Swingle Leaf Extracts Obtained by Two-Step Sequential Extraction. Antioxidants.

[74] Cselőtey A., Baglyas M., Király N., Ott P.G., Glavnik V., Vovk I., Móricz Á.M. (2024). Bioassay-Guided Isolation and Identification of Antibacterial Compounds from Invasive Tree of Heaven Stem and Trunk Bark. Molecules.

[75] Weidner C., De Groot J.C., Prasad A., Freiwald A., Quedenau C., Kliem M., Witzke A., Kodelja V., Han C.-T., Giegold S. (2012). Amorfrutins are potent antidiabetic dietary natural products. Proc. Natl. Acad. Sci. USA.

[76] Kim Y.S., Ryu Y.B., Curtis-Long M.J., Yuk H.J., Cho J.K., Kim J.Y., Kim K.D., Lee W.S., Park K.H. (2011). Flavanones and rotenoids from the roots of *Amorpha fruticosa* L. that inhibit bacterial neuraminidase. Food Chem. Toxicol..

[77] Wu X., Liao H.-B., Li G.-Q., Liu Y., Cui L., Wu K.-F., Zhu X.-H., Zeng X.-B. (2015). Cytotoxic rotenoid glycosides from the seeds of *Amorpha fruticosa*. Fitoterapia.

[78] Cui X., Guo J., Lai C.-S., Pan M.-H., Ma Z., Guo S., Liu Q., Zhang L., Ho C.-T., Bai N. (2017). Analysis of bioactive constituents from the leaves of *Amorpha fruticosa* L.. J. Food Drug Anal..

[79] Lee W., Yoon G., Kim M.C., Kwon H.C., Bae G.-U., Kim Y.K., Kim S.-N. (2016). 5,7-Dihydroxy-6-geranylflavanone improves insulin sensitivity through PPARα/γ dual activation. Int. J. Mol. Med..

[80] Jankovská D., Jurčová N., Kubínová R., Václavík J., Švajdlenka E., Mascellani A., Maršík P., Bouzková K., Malaník M. (2024). Anticholinesterase Activity of Methanolic Extract of *Amorpha fruticosa* Flowers and Isolation of Rotenoids and Putrescine and Spermidine Derivatives. Plants.

[81] Schlick J.-L., Dulieu P., Desvoyes B., Adami P., Radom J., Jouvenot M. (2000). Cytotoxic activity of a recombinant GnRH-PAP fusion toxin on human tumor cell lines. FEBS Lett..

[82] Ishag H.Z.A., Li C., Huang L., Sun M., Ni B., Guo C., Mao X. (2013). Inhibition of Japanese encephalitis virus infection in vitro and in vivo by pokeweed antiviral protein. Virus Res..

[83] Mansouri S., Choudhary G., Sarzala P.M., Ratner L., Hudak K.A. (2009). Suppression of Human T-cell Leukemia Virus I Gene Expression by Pokeweed Antiviral Protein. J. Biol. Chem..

[84] Zhabokritsky A., Mansouri S., Hudak K.A. (2014). Pokeweed antiviral protein alters splicing of HIV-1 RNAs, resulting in reduced virus production. RNA.

[85] Takahasi H., Yanagi K., Ueda M., Nakade K., Fukuyama Y. (2003). Structures of 1,4-Benzodioxane Derivatives from the Seeds of *Phytolacca americana* and Their Neuritogenic Activity in Primary Cultured Rat Cortical Neurons. Chem. Pharm. Bull..

[86] Zheleva-Dimitrova D. (2013). Antioxidant and acetylcholinesterase inhibition properties of *Amorpha fruticosa* L. and *Phytolacca americana* L.. Pharmacogn. Mag..

[87] Patra J.K., Kim E.S., Oh K., Kim H.-J., Kim Y., Baek K.-H. (2014). Antibacterial effect of crude extract and metabolites of *Phytolacca americana* on pathogens responsible for periodontal inflammatory diseases and dental caries. BMC Complement. Altern. Med..

[88] Saleri F.D., Chen G., Li X., Guo M. (2017). Comparative Analysis of Saponins from Different Phytolaccaceae Species and Their Antiproliferative Activities. Molecules.

[89] Popovici L.-F., Brinza I., Gatea F., Badea G.I., Vamanu E., Oancea S., Hritcu L. (2025). Enhancement of Cognitive Benefits and Anti-Anxiety Effects of *Phytolacca americana* Fruits in a Zebrafish (*Danio rerio*) Model of Scopolamine-Induced Memory Impairment. Antioxidants.

[90] Aslam B., Asghar R., Muzammil S., Shafique M., Siddique A.B., Khurshid M., Ijaz M., Rasool M.H., Chaudhry T.H., Aamir A. (2024). AMR and Sustainable Development Goals: At a crossroads. Glob. Health.

[91] Bhojyawal V., Kesarwani M., Gupta S., Gangwar M., Nath G. (2024). The Rise of Antibiotic Resistance: A Global Threat, Origin, and Evolution of Antibiotic Resistance: Current Scenario and Future Prospective. Emerging Paradigms for Antibiotic-Resistant Infections: Beyond the Pill.

[92] Ahmed S.K., Hussein S., Qurbani K., Ibrahim R.H., Fareeq A., Mahmood K.A., Mohamed M.G. (2024). Antimicrobial resistance: Impacts, challenges, and future prospects. J. Med. Surg. Public Health.

[93] Muteeb G., Rehman M.T., Shahwan M., Aatif M. (2023). Origin of Antibiotics and Antibiotic Resistance, and Their Impacts on Drug Development: A Narrative Review. Pharmaceuticals.

[94] Porras G., Chassagne F., Lyles J.T., Marquez L., Dettweiler M., Salam A.M., Samarakoon T., Shabih S., Farrokhi D.R., Quave C.L. (2021). Ethnobotany and the Role of Plant Natural Products in Antibiotic Drug Discovery. Chem. Rev..

[95] Murugaiyan J., Kumar P.A., Rao G.S., Iskandar K., Hawser S., Hays J.P., Mohsen Y., Adukkadukkam S., Awuah W.A., Jose R.A.M. (2022). Progress in Alternative Strategies to Combat Antimicrobial Resistance: Focus on Antibiotics. Antibiotics.

[96] Chandra H., Bishnoi P., Yadav A., Patni B., Mishra A.P., Nautiyal A.R. (2017). Antimicrobial Resistance and the Alternative Resources with Special Emphasis on Plant-Based Antimicrobials—A Review. Plants.

[97] Arip M., Selvaraja M., Rajagopal M., Tan L.F., Leong M.Y., Tan P.L., Yap V.L., Chinnapan S., Tat N.C., Abdullah M. (2022). Review on Plant-Based Management in Combating Antimicrobial Resistance—Mechanistic Perspective. Front. Pharmacol..

[98] Atta S., Waseem D., Fatima H., Naz I., Rasheed F., Kanwal N. (2023). Antibacterial potential and synergistic interaction between natural polyphenolic extracts and synthetic antibiotic on clinical isolates. Saudi J. Biol. Sci..

[99] Alam M., Bano N., Ahmad T., Sharangi A.B., Upadhyay T.K., Alraey Y., Alabdallah N.M., Rauf M.A., Saeed M. (2022). Synergistic Role of Plant Extracts and Essential Oils against Multidrug Resistance and Gram-Negative Bacterial Strains Producing Extended-Spectrum β-Lactamases. Antibiotics.

[100] Ramata-Stunda A., Petriņa Z., Valkovska V., Borodušķis M., Gibnere L., Gurkovska E., Nikolajeva V. (2022). Synergistic Effect of Polyphenol-Rich Complex of Plant and Green Propolis Extracts with Antibiotics against Respiratory Infections Causing Bacteria. Antibiotics.

[101] Jin X., Ren J., Li R., Gao Y., Zhang H., Li J., Zhang J., Wang X., Wang G. (2021). Global burden of upper respiratory infections in 204 countries and territories, from 1990 to 2019. eClinicalMedicine.

[102] Pandey A., Galvani A.P. (2019). The global burden of HIV and prospects for control. Lancet HIV.

[103] Michaud C.M. (2009). Global Burden of Infectious Diseases. Encycl. Microbiol..

[104] Wong Y., Meehan M.T., Burrows S.R., Doolan D.L., Miles J.J. (2022). Estimating the global burden of Epstein–Barr virus-related cancers. J. Cancer Res. Clin. Oncol..

[105] Ali S.I., Sheikh W.M., Rather M.A., Venkatesalu V., Muzamil Bashir S., Nabi S.U. (2021). Medicinal plants: Treasure for antiviral drug discovery. Phytother. Res..

[106] Birben E., Sahiner U.M., Sackesen C., Erzurum S., Kalayci O. (2012). Oxidative Stress and Antioxidant Defense. World Allergy Organ. J..

[107] Kıran T.R., Otlu O., Karabulut A.B. (2023). Oxidative stress and antioxidants in health and disease. J. Lab. Med..

[108] Pandhair V., Sekhon B.S. (2006). Reactive Oxygen Species and Antioxidants in Plants: An Overview. J. Plant Biochem. Biotechnol..

[109] Ahmad P., Sarwat M., Sharma S. (2008). Reactive oxygen species, antioxidants and signaling in plants. J. Plant Biol..

[110] Kotha R.R., Tareq F.S., Yildiz E., Luthria D.L. (2022). Oxidative Stress and Antioxidants—A Critical Review on In Vitro Antioxidant Assays. Antioxidants.

[111] López-Alarcón C., Denicola A. (2013). Evaluating the antioxidant capacity of natural products: A review on chemical and cellular-based assays. Anal. Chim. Acta.

[112] Ling T., Lang W.H., Maier J., Quintana Centurion M., Rivas F. (2019). Cytostatic and Cytotoxic Natural Products against Cancer Cell Models. Molecules.

[113] Fridlender M., Kapulnik Y., Koltai H. (2015). Plant derived substances with anti-cancer activity: From folklore to practice. Front. Plant Sci..

[114] Cragg G.M., Newman D.J. (2005). Plants as a source of anti-cancer agents. J. Ethnopharmacol..

[115] Dehelean C.A., Marcovici I., Soica C., Mioc M., Coricovac D., Iurciuc S., Cretu O.M., Pinzaru I. (2021). Plant-Derived Anticancer Compounds as New Perspectives in Drug Discovery and Alternative Therapy. Molecules.

[116] Garcia-Oliveira P., Otero P., Pereira A.G., Chamorro F., Carpena M., Echave J., Fraga-Corral M., Simal-Gandara J., Prieto M.A. (2021). Status and Challenges of Plant-Anticancer Compounds in Cancer Treatment. Pharmaceuticals.

[117] Seca A.M.L., Pinto D.C.G.A. (2018). Plant Secondary Metabolites as Anticancer Agents: Successes in Clinical Trials and Therapeutic Application. Int. J. Mol. Sci..

[118] ALNasser M.N., Alboraiy G.M., Alsowig E.M., Alqattan F.M. (2025). Cholinesterase Inhibitors from Plants and Their Potential in Alzheimer’s Treatment: Systematic Review. Brain Sci..

[119] dos Santos T.C., Gomes T.M., Pinto B.A.S., Camara A.L., de Andrade Paes A.M. (2018). Naturally Occurring Acetylcholinesterase Inhibitors and Their Potential Use for Alzheimer’s Disease Therapy. Front. Pharmacol..

[120] Ahmed F., Ghalib R.M., Sasikala P., Ahmed K.K.M. (2013). Cholinesterase inhibitors from botanicals. Pharmacogn. Rev..

